# Cannabinoid Signaling in the Skin: Therapeutic Potential of the “C(ut)annabinoid” System

**DOI:** 10.3390/molecules24050918

**Published:** 2019-03-06

**Authors:** Kinga Fanni Tóth, Dorottya Ádám, Tamás Bíró, Attila Oláh

**Affiliations:** 1 Department of Physiology, Faculty of Medicine, University of Debrecen, 4032 Debrecen, Hungary; toth.kinga.fanni@med.unideb.hu (K.F.T.); adam.dorottya@med.unideb.hu (D.Á.); 2 Department of Immunology, Faculty of Medicine, University of Debrecen, 4032 Debrecen, Hungary; biro.tamas@med.unideb.hu; 3 HCEMM Nonprofit Ltd., 6720 Szeged, Hungary

**Keywords:** acne, atopic dermatitis, cannabinoid, fibrosis, hair growth, inflammation, itch, psoriasis, skin, tumor, wound healing

## Abstract

The endocannabinoid system (ECS) has lately been proven to be an important, multifaceted homeostatic regulator, which influences a wide-variety of physiological processes all over the body. Its members, the endocannabinoids (eCBs; e.g., anandamide), the eCB-responsive receptors (e.g., CB_1_, CB_2_), as well as the complex enzyme and transporter apparatus involved in the metabolism of the ligands were shown to be expressed in several tissues, including the skin. Although the best studied functions over the ECS are related to the central nervous system and to immune processes, experimental efforts over the last two decades have unambiguously confirmed that cutaneous cannabinoid (“c[ut]annabinoid”) signaling is deeply involved in the maintenance of skin homeostasis, barrier formation and regeneration, and its dysregulation was implicated to contribute to several highly prevalent diseases and disorders, e.g., atopic dermatitis, psoriasis, scleroderma, acne, hair growth and pigmentation disorders, keratin diseases, various tumors, and itch. The current review aims to give an overview of the available skin-relevant endo- and phytocannabinoid literature with a special emphasis on the putative translational potential, and to highlight promising future research directions as well as existing challenges.

## 1. Introduction

### 1.1. The Barrier and Beyond: Novel Aspects of Cutaneous (Patho)physiology

The skin is a vital organ that fulfills multiple roles. Besides being a complex protective barrier against a wide-variety of environmental challenges [1,2,3], it is an active neuroendocrinoimmuno organ, which produces several hormones, plays an important role in thermoregulation, and is involved in the detection of various environmental signals, as well as in their translation/transmission to the nervous and immune systems [3,4,5]. Indeed, functional expression of olfactory [6,7], photo [8,9], and taste receptors [10,11,12]—among others—has recently been proven in different non-neuronal cells of the integumentary system.

The complex protection provided by the skin is based on a fine-tuned barrier system, which includes the cutaneous physicochemical, immunological and microbiological barriers. The development of this complex barrier requires active and tightly regulated cooperation, and therefore appropriate communication of several cell types, including numerous “professional” immune cells (e.g., Langerhans cells, dendritic cells, macrophages, mast cells, various T cell populations), and other cell types (e.g., keratinocytes, fibroblasts, melanocytes, sebocytes, adipocytes) [1,2,3,13,14,15,16,17,18]. Moreover, cells of the human skin express a wide-array of pathogen- and danger-associated molecular pattern recognizing receptors, and are capable of producing several anti-microbial peptides and lipids, as well as pro- and anti-inflammatory cytokines and chemokines, by which they can initiate and regulate local immune responses [1,2,4,16,17,18,19,20,21,22,23,24,25]. Obviously, these interactions are under the tight control of several signaling systems, among which the current review aims to focus on a remarkably multifaceted one, namely the cutaneous cannabinoid (“c[ut]annabinoid”) system.

### 1.2. (Endo)cannabinoid Signaling and its most Important Interactions

**Figure 1:**
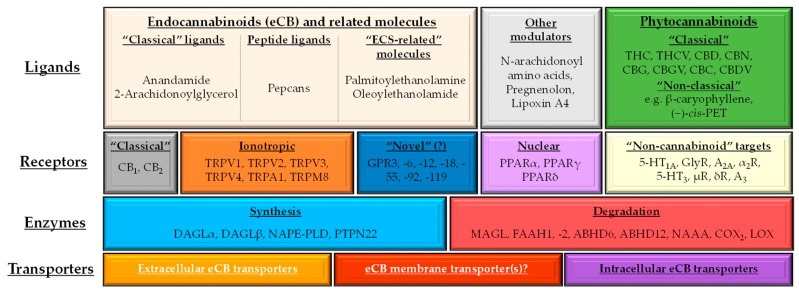
Schematic overview of the (endo)cannabinoid system (ECS) and its putative connections to other signaling systems. Depending on how we choose to limit the definition, the number of the putative ligands as well as that of the possible targets increases dramatically; therefore, on the figure, we only summarize the most important ones. Each ligand possesses a unique molecular fingerprint, i.e., the ability to concentration-dependently activate/antagonize/inhibit a selected group of possible targets. Obviously, all these actions are highly context-dependent (e.g., they are influenced by the relative expression of the potential targets in the given tissue, the concentration of the substance), resulting in characteristic, and in some cases even opposing biological responses. Although the classical, lipophilic eCBs definitely require inter- and intracellular carriers, relatively little is known about these transporter systems. Intracellular eCB transporters may include fatty acid binding proteins (FABPs) and heat shock protein 70 (HSP70), whereas FABP4, albumins, HSP70 and extracellular vesicles [61,62] are likely to be involved in their intercellular transport [63]. With respect to FAAH1 and -2 it is important to note that only scarce evidence is available about the expression and functionality of the latter. Intriguingly, FAAH2 is not expressed in mice and rats, but shares substrate spectrum of FAAH1 (however, it has inferior affinity towards AEA and N-acyl taurines). Conventional FAAH-inhibitors can inhibit its activity [48], and its missense polymorphism (A458S) may lead to psychiatric disorders (anxiety, mild learning disability) [64]. Later in the text, except when stated otherwise, by mentioning “FAAH”, we refer to “FAAH1”. 5-HT: 5-hydroxytryptamine (serotonin) receptor; A_2A_ and A_3_: adenosine 2A and 3 receptors; ABDH6 and -12: α/β-hydrolase domain containing 6 and 12; CBC: (−)-cannabichromene; CBD: (−)-cannabidiol; CBDV: (−)-cannabidivarin; CBG: (−)-cannabigerol; CBGV: (−)-cannabigerovarin; CBN: (−)-cannabinol; (−)-*cis*-PET: (−)-*cis*-perrottetinene; COX_2_: cyclooxygenase-2; DAGL: diacylglycerol lipase; eCB: endocannabinoid; FAAH: fatty acid amide hydrolase; GPR: G protein-coupled receptor; LOX: lipoxygenase; MAGL: monoacylglycerol lipase; NAAA: N-acylethanolamine hydrolyzing acid amidase; NAPE-PLD: N-acylphosphatidylethanolamine-specific phospholipase D; PPAR: peroxisome proliferator-activated receptor; PTPN22: protein tyrosine phosphatase non-receptor type 22; THC: (−)-*trans*-Δ^9^-tetrahydrocannabinol; THCV: (−)-Δ^9^-tetrahydrocannabivarin; TRP: transient receptor potential.

The endocannabinoid system (ECS) is a complex, evolutionarily conserved [26,27,28,29,30] homeostatic signaling network. It comprises endogenous ligands (endocannabinoids [eCB], e.g., anandamide [AEA]), eCB-responsive receptors (e.g., CB_1_ and CB_2_ cannabinoid receptors), and a complex enzyme and transporter apparatus. These molecules are involved in the synthesis (e.g., N-acyl phosphatidylethanolamine-specific phospholipase D [NAPE-PLD], diacylglycerol lipase [DAGL]-α and -β, protein tyrosine phosphatase non-receptor type 22 [PTPN22]), cellular uptake and release (i.e., the putative endocannabinoid membrane transporter(s) [EMT]), inter- and intracellular transport (e.g., fatty acid binding proteins), and degradation (e.g., fatty acid amide hydrolase [FAAH], monoacylglycerol lipase [MAGL]) of eCBs (Figure 1) [31,32,33,34,35,36,37,38,39,40,41,42,43,44,45,46,47,48,49,50]. Importantly, depending on the definition, several other endogenous molecules can be classified as “cannabinoid-like” or “cannabinoid-related” (e.g., palmitoylethanolamine [PEA], oleoylethanolamide [OEA]) beyond the “classical” eCBs [31,32,33,34,35,36,37,38,39,40,41,42,43,44,45,46,47,51].

Besides eCBs and related endogenous mediators, the *Cannabinaceae*-derived “classical” (e.g., the psychotropic (−)-*trans*-Δ^9^-tetrahydrocannabinol [THC] or the non-psychotropic (−)-cannabidiol [CBD]) and other plants-derived “non-classical” (e.g., the CB_2_-selective agonist β-caryophyllene, or the liverwort-derived (−)-*cis*-perrottetinene [(−)-*cis*-PET]) phytocannabinoids (pCBs) represent another important, and ever growing group of cannabinoids [31,32,33,34,35,36,37,38,39,40,41,42,43,44,45,46,47,52]. To date, more than 500 biologically active components were identified in the plants of the *Cannabis* genus, among which more than 100 were classified as pCBs. Moreover, as mentioned above, several other plants were already shown to produce molecules with cannabinoid activity [30,32,47,52]. It is suggested that consumption of cannabimimetic food components might have played a role in hominid evolution, and production of cannabimimetic food seems to be a promising future nutraceutical strategy [30].

**Figure 2:**
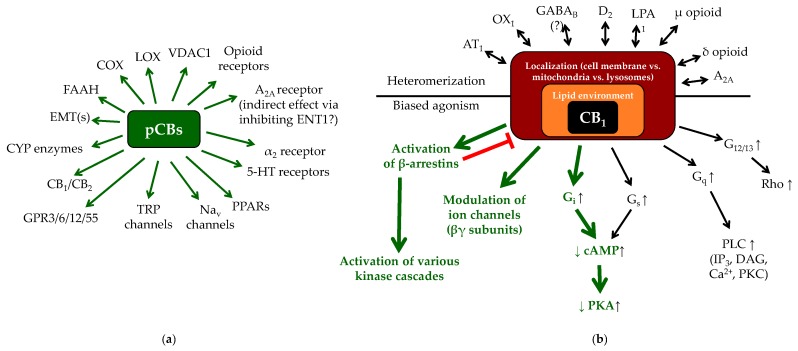
Examples of the context-dependent complexity of the cannabinoid signaling. (**a**) Overview of the most important potential targets of the phytocannabinoids (pCBs), which can be concentration-dependently activated/antagonized/inhibited by these molecules. Each pCB can be characterized by a unique molecular fingerprint, and every pCB was found to interact with only a subset of potential targets shown on panel (**a**). Importantly, the interactions can even result in opposing outcomes (e.g., THC is a partial CB_1_ agonist, whereas CBD is a CB_1_ antagonist/inverse agonist), making prediction of cellular effects of the pCBs even more difficult. (**b**) The actual biological response, which develops following the activation of CB_1_ receptor depends on several additional factors, including biased agonism [31,32,65,66,67,68,69,70,71,72,73], possible receptor heteromerization [32,74,75,76,77,78,79,80], localization (i.e., cell membrane vs. mitochondria vs. lysosomes [81,82,83]), as well as the composition of the lipid microenvironment of the given membrane [58,84]. Green arrows on panel (b): the most common signaling pathways of CB_1_. Note that besides CB_1_, biased agonism is well-described in case of CB_2_, GPR18, GPR55 and GPR119 as well, whereas CB_2_ was proven to heteromerize with, e.g., C-X-C chemokine receptor type 4 chemokine receptor (CXCR4), or GPR55 (for details, see the above references). The question mark indicates that functional heteromerization of CB_1_ and GABA_B_ receptors is questionable. AT_1_: angiotensin II receptor type 1; CYP: cytochrome P450 enzymes; D_2_: dopamine receptor 2; EMT(s): endocannabinoid membrane transporter(s); ENT1: equilibrative nucleoside transporter 1; GABA_B_: γ-aminobutyric acid receptor B; LPA_1_: lysophosphatidic acid receptor 1; Na_v_: voltage-gated Na^+^ channels; OX_1_: orexin 1 receptor; VDAC1: voltage-dependent anion channel 1. The figure was adapted and modified from [31] originally licensed under CC-BY, version 4.0.

Depending on their concentration, eCBs and pCBs are able to activate/antagonize/inhibit a remarkably wide-variety of cellular targets including several metabotropic (e.g., CB_1_ or CB_2_), ionotropic (certain transient receptor potential [TRP] ion channels) and nuclear (peroxisome proliferator-activated receptors [PPARs]) receptors, various enzymes, and transporters [31,32,33,34,35,36,37,38,39,40,41,42,43,44,45,46,47,53,54,55,56] (Figure 1). Importantly, each ligand can be characterized by a unique, molecular fingerprint, and in some cases, they can even exert opposing biological actions on the same target molecule (Figure 2a).

Indeed, it was nicely shown in several biochemical studies that THC was a partial CB_1_ agonist, whereas CBD was an antagonist/inverse agonist of the receptor [57]. Keeping this in mind it is easy to understand why CBD is co-administered with THC in the oromucosal spray Sativex^®^, where the intent is to prevent the onset of potential psychotropic side effects rooting from the THC-induced activation of CB_1_ expressed in the central nervous system [58]. Intriguingly, despite solid experimental and clinical evidence proving that CBD is able to antagonize CB_1_, it is very important to emphasize that it can *context-dependently* behave as a *functional CB*_1_ *activator* as well. Indeed, by inhibiting FAAH and/or EMT, its administration can lead to an elevation of the local eCB-tone, and hence to an indirectly increased CB_1_ activity in certain systems [59,60].

The high number of possible ligands and cellular targets together with the above context-dependence already indicate that one has to be very careful when predicting the biological effects of each cannabinoid based on mere biochemical observations obtained in artificially “clean” overexpressor systems. Still, use and systematic assessment of such systems is extremely important because of additional layers of complexity in (endo)cannabinoid signaling, including signaling bias (i.e., ligand-dependent preference to the second messenger system) [31,32,65,66,67,68,69,70,71,72,73], receptor heteromerization [32,74,75,76,77,78,79,80], cellular localization (surface membrane, mitochondria [81,82] or lysosomes [83]), the regulatory role of the membrane lipid microenvironment [58,84] or agonist-induced down-regulation [85] (Figure 2b). Finally, in some cases, effects of non-conventional activators should also be taken into consideration, since certain cannabinoid-responsive receptors (namely CB_1_, CB_2_, and TRPV4) were shown to be activated by UV-irradiation as well [86,87].

### 1.3. Cannabinoids in the Skin: Brief Overview of the “c(ut)annabinoid” Signaling

It has recently been shown that abuse of synthetic, hyperpotent cannabinoids (e.g., “Bonsai”, “fake weed”, “K2”, and “Jamaica”) can result in dermatological disorders, such as premature skin aging, hair loss and graying, or acne [88], indicating that cannabinoid signaling can profoundly influence skin biology. Indeed, several lines of evidence demonstrate that both endogenous and phytocannabinoids can exert various biological effects in the skin, implicating cannabinoid signaling as a key contributor to cutaneous homeostasis. The presence of different eCBs, cannabinoid receptors, as well as other members of the ECS has already been shown on many different cell types of the skin, including, but not limited to epidermal keratinocytes, melanocytes, mast cells, fibroblasts, sebocytes, sweat gland cells, as well as certain cell populations of hair follicles. Since these data have been extensively reviewed in excellent recent papers [88,89,90,91,92,93,94,95,96,97,98,99,100,101], besides providing a brief general overview, our current paper intends to focus on areas which have received less attention in said papers, and to highlight the mostly neglected therapeutic potential present in the pharmacological modulation of the “c(ut)annabinoid” signaling. Last, but not least, we intend to discuss the potential limitations and side effects of such medications as well.

## 2. Translational Potential of the Cutaneous Cannabinoid Signaling

### 2.1. Sebaceous Gland (SG)-Related Disorders: Acne and Skin Dryness

The most obvious role of sebaceous glands (SG) is the production of lipid-rich sebum, which contributes to the development of the physicochemical barrier, and, via its acid and anti-microbial lipid content, also controls the growth of cutaneous microbiota [102,103,104]. SGs have endocrine and immune regulatory functions as well [105,106,107,108,109], and their clinical significance is also very high, since they are key players in the pathogenesis of highly prevalent dermatoses such as acne and seborrhea, and their dysfunction contributes to the development of dryness-accompanied skin diseases, including atopic dermatitis (AD) [102,103,105,110].

The clinical observation that cannabinoid abuse can be accompanied by acne, already highlights how cannabinoid signaling may influence human sebocyte biology [88]. Indeed, expression of CB_1_ (in the differentiated, central cells) and CB_2_ (predominantly in the basal, non-differentiated sebocytes) receptors in human SGs was first demonstrated by Ständer and her co-workers in 2005 [111]. When exploring the functional relevance of these findings, it has been shown that CB_2_ is likely to contribute to the maintenance of homeostatic sebaceous lipogenesis (SLG), since siRNA-mediated silencing of the receptor significantly decreased lipid production, whereas administration of AEA and 2-AG (30 μM) led to excessive lipid synthesis via the activation of a CB_2_→ERK1/2 MAPK→PPAR pathway [112]. Later on, the major eCB synthesizing (NAPE-PLD, DAGLα and −β) and degrading (MAGL and FAAH) enzymes were found to be expressed both in cultured human immortalized SZ95 sebocytes [108,109,113] and in situ in human SGs, with the sole exception of DAGLα [114], the expression of which was observed to be much weaker as compared to the endogenous tissue positive control [115] sweat glands.

It has also been demonstrated that certain EMT-inhibitors (VDM11 and AM404), but, intriguingly, not the FAAH-inhibitor URB597, promoted SLG, and VDM11-induced elevation of the eCB-tone suppressed the pro-inflammatory action of the Toll-like receptor (TLR)-4 activator lipopolysaccharide (LPS) [114]. Considering that, as mentioned above, SG hypoplasia and dysfunction contributes to the development of dryness-accompanied skin diseases [102,103], and that such diseases often have inflammatory components, a moderate (i.e., not excessive, seborrheic/acnegenic) elevation of physiological SLG together with the suppression of the release of pro-inflammatory cytokines and chemokines could exert beneficial effects. Thus, the available data [114] highlight the possibility that eCB transport inhibitors might have beneficial effects in diseases with skin dryness such as AD. Future studies are therefore invited to explore the exact impact of VDM11 treatment on the sebaceous lipidome to reduce the possibility of potential acnegenic side effects.

Interestingly, that study also demonstrated that human sebocytes were involved in the metabolism of PEA and OEA [114]. Moreover, the expression of an important cellular target of the latter, namely GPR119, was also identified on human sebocytes. The available scarce evidence suggests that the OEA→GPR119→ERK1/2 MAPK signaling chain may be a previously unknown promoter of sebocyte differentiation, and therefore dysregulation of this pathway may contribute to the development of seborrhea and acne [116]. This seems to be particularly interesting, since GPR119 has recently emerged as a promising therapeutic target in type 2 diabetes mellitus. Although the tested synthetic agonists have not passed yet phase II clinical trials [117], and both endogenous and synthetic agonists of GPR119 may exhibit biologically relevant signaling bias [73], these preliminary findings warn of the risk of unexpected cutaneous side effects upon administration of GPR119 activators exhibiting “OEA-like” signaling preference [116].

Intriguingly, besides the aforementioned “classical” members of the ECS, functional expression of several ECS-related TRP channels was also demonstrated. The mostly Ca^2+^-permeable ion channels TRPV1, TRPV2, TRPV3 and TRPV4 [118,119,120] were shown to be expressed on human sebocytes. Importantly, in a striking contrast to the “classical” cannabinoid signaling, activation of the TRPV channels was proven to decrease SLG. Moreover, activation of TRPV3 led to a significant pro-inflammatory response in the sebocytes as revealed by the up-regulated expression and increased release of several pro-inflammatory cytokines [119], a phenomenon recently demonstrated on human epidermal keratinocytes as well [121].

Notably, the best-studied non-psychotropic pCB, i.e., CBD (10 μM), was found to exert complex anti-acne effects by normalizing several pro-acne agents-induced excessive SLG, and by exerting anti-proliferative and anti-inflammatory actions, without influencing homeostatic SLG or viability of human sebocytes. Importantly, the lipostatic and anti-proliferative effects were found to be mediated by the TRPV4→[Ca^2+^]_IC_↑→ERK1/2 MAPK↓ and nuclear receptor interacting protein 1 (NRIP1, a.k.a. RIP140)↓ signaling pathway, whereas the anti-inflammatory actions were coupled to the (most likely indirect) activation of the adenosine A_2A_ receptor→cAMP↑→tribbles homolog 3 (TRIB3)↑→p65-NF-κB↓ pathway [120]. This, together with the fact that CBD was shown to suppress proliferation [122] and differentiation [59] of human keratinocytes, and to exert potent anti-bacterial effects [123], collectively argue that it may be an efficient anti-acne agent in vivo as well.

This concept was further supported by a small, single-blind, split-face study, in which a cream containing 3% *Cannabis* seed extract was applied twice daily to the cheeks of patients for 12 weeks. The treatment was found to be efficient in reducing sebum production and erythema compared to the vehicle treated side [124]. Moreover, a synthetic CBD containing special topical formulation (“BTX 1503”) exhibited promising anti-acne potential in a small phase Ib clinical trial [125], and its efficacy is now being tested in a randomized, double-blind, vehicle-controlled phase II clinical study (ClinicalTrials.gov ID: NCT03573518).

Last, but not least, it should also be noted that effects of several other non-psychotropic pCBs, namely CBC, CBDV, CBG, CBGV and THCV were also assessed in human sebocytes. This latter study found an intriguing functional heterogeneity between the tested pCBs, with CBC, CBDV and most especially THCV behaving in a “CBD-like” manner (potent complex anti-acne effects in vitro), whereas CBG and CBGV being more “eCB-like” substances (slight, but significant promotion of SLG together with potent anti-inflammatory activity) [126]. Although the exact impact of CBG and CBGV on the sebaceous lipidome remains to be tested in future studies, the available evidence suggests that, similar to the aforementioned EMT-inhibitors VDM11 and AM404, they might have therapeutic value in dryness- and inflammation-accompanied skin diseases. The putative SG-related translational potential of cannabinoid signaling is summarized in Table 1.

### 2.2. Hair Growth Disorders: Alopecia, Effluvium, Hirsutism, Hypertrichosis

Hair follicles (HF) are unique miniorgans of the human body. They exhibit immune privilege (IP), i.e., they can be characterized by low or absent major histocompatibility complex (MHC) class Ia and β2 microglobulin expression leading to an ineffective self-peptide presentation, and they secrete several immunosuppressants to create an immunoinhibitory milieu [127,128]. Besides this, HFs are characterized by life-long cycles of growing (anagen), regressive (catagen) and “quasi-quiescent” (telogen) life phases collectively referred to as the “hair cycle” [129]. Importantly, dysregulation of this cycle (e.g., premature termination or abnormal prolongation of the anagen phase) lies at the base of several clinically important hair growth disorders leading to unwanted hair loss (i.e., various alopecia forms) or undesired hair growth (hirsutism and hypertrichosis).

Similar to SGs, the biology of HFs is also influenced by cannabinoids. Indeed, as mentioned above, abuse of certain synthetic cannabinoids was shown to result in hair loss and graying [88], and it is well-proven that CB_1_ is expressed in human HFs, whereas regarding the expression of CB_2_ contradictory findings have been published so far [111,130,131,132,133]. Of great importance, prototypic eCBs (i.e., AEA and 2-AG) were shown to be produced in human HFs, among which 30 μM AEA (but not 2-AG) was proven to inhibit hair growth by inducing premature catagen entry in a CB_1_-dependent manner, but, somewhat surprisingly, it did not influence the pigmentation of HFs.

In line with these findings, 2–20 μM THC was also shown to inhibit hair shaft elongation, and to induce catagen entry, but, unlike AEA, it also suppressed melanogenesis in anagen VI HFs, highlighting an intriguing functional heterogeneity between cannabinoids, which might have reflected the aforementioned (Section 1.2, Figure 2b) signaling bias of the tested compounds. Importantly, CB_1_ itself was greatly up-regulated in the hair matrix keratinocytes both in AEA- and interferon-γ (IFN-γ)-induced catagen, supporting the concept that it may play a role in the termination of the HF growth phase [130]. The idea that CB_1_ is a negative regulator of HF growth was further supported by animal data. Indeed, an orally administered rimonabant analogue CB_1_ antagonist (“compound 3”) promoted hair growth (and had antiobesity effects) in C57BL/6J mice, in which high fat diet induced obesity was accompanied by alopecia. Interestingly, however, the effect of the CB_1_ antagonist did not develop if it was applied topically [134].

Besides CB_1_, several cannabinoid-responsive TRPV channels (namely TRPV1, TRPV3 and TRPV4) were shown to be functionally expressed in human HFs, and to promote the onset of catagen phase [135,136,137,138], which, considering that all three channels are heat-sensitive [45,94,95,139], may be an evolutionary relic of warmth-induced shedding. Last, but not least, preliminary evidence suggests that CBD may concentration-dependently promote (0.1 μM) or suppress (10 μM) hair shaft elongation, most likely in adenosine receptor and TRPV4-dependent manners, respectively [140].

Finally, considering the well-known anti-inflammatory and immunosuppressive effects of cannabinoids [31,33,93,141,142,143,144,145], it is not surprising that certain data suggest involvement of cannabinoid dysregulation in the development of alopecia areata (AA). AA is an autoimmune disease characterized by localized or global hair loss due to the collapse of the HF IP and the subsequent autoaggression of cytotoxic T cells leading to premature catagen entry. Importantly, several lines of evidence suggest that a loss-of-function single-nucleotide polymorphism (C1858T substitution; “R620W variant”; “rs2476601”) of PTPN22 (a phosphatase involved in synthesizing AEA [48], which normally suppresses T-cell proliferation), which leads to its rapid degradation, is coupled to several autoimmune diseases (for details, see [31]), including alopecia areata (AA) [146,147,148,149,150,151,152]. Although PTPN22 has several other functions besides AEA synthesis [153], and eCB levels were not measured yet in lesional skin of AA patients, one might hypothesize based on the above correlation that a decrease in the anti-inflammatory eCB-tone induced by PTPN22 dysfunction might contribute to the onset of the disease. Thus, elevation of the eCB-tone as well as direct CB_1_ agonism might be promising tools to prevent the onset/relapse of AA. Finally, albeit only scant evidence is available, it is noteworthy that some experimental [140] and pilot clinical data [154] highlight the possibility that carefully selected doses of topically applied CBD might also exert beneficial effects in AA. Further studies, as well as well-controlled clinical trials are therefore invited to elucidate the putative therapeutic potential of the cutaneous cannabinoid and related signaling systems in AA. Putative hair-related translational potential of the cannabinoid signaling is summarized in Table 2.

### 2.3. Melanocytes & Pigmentation Disorders

Primary human melanocytes were shown to produce AEA and 2-AG [155], and to express GPR119 (only mRNA data) [156], CB_1_, CB_2_ and TRPV1 together with NAPE-PLD, DAGL, FAAH and MAGL [155]. However, expression of MAGL in normal human epidermal melanocytes was questioned in a recent study stating that this enzyme was only expressed in melanoma cells, where its expression correlated with the aggressiveness of the tumor [157].

In functional studies, 100–150 μM β-caryophyllene was found to inhibit spontaneous melanogenesis of mouse B16 melanoma cells [158], whereas 5 μM AEA was shown to induce apoptosis of primary human melanocytes most likely by activating TRPV1. Lower (≤3 μM) AEA concentrations however, dose-dependently stimulated melanogenesis and tyrosinase activity in a CB_1_-dependent manner through the activation of p38 and ERK1/2 MAPK, as well as the cAMP response element-binding protein (CREB), but without influencing the cAMP level [155].

In line with these observations, CBD was also shown to enhance melanogenesis and tyrosinase activity of primary human epidermal melanocytes by (most probably indirectly) activating the same CB_1_-coupled signaling pathway [60]. Although these data argue that CB_1_ agonism may be a potent tool to treat hypopigmentation, other findings suggest that the overall effects of the eCB-signaling might be more complex. Indeed, by using co-cultures of a human melanotic melanoma cell line (SK-mel-1) and HaCaT keratinocytes (a spontaneously immortalized human epidermal keratinocyte cell line [159]), Magina and her co-workers found that CB_1_ agonism reduced both spontaneous and UVB-induced melanogenesis, highlighting that the local tissue microenvironment may have an important role in regulating melanocyte functions [160]. Finally, in contrast to AEA, OEA (10–50 μM) was shown to markedly inhibit melanin synthesis and tyrosinase activity in α-MSH-stimulated B16 mouse melanoma cells in a PPARα-independent manner. Its effects were found to be coupled to the activation of p38 and ERK1/2 MAPK, as well as of Akt signaling cascades, and inhibition of the CREB pathway (unfortunately, putative involvement of GPR119 was not assessed) [161]. Thus, (endo)cannabinoid signaling appears to exert a complex regulatory role in melanocytes; however, the results are greatly model-dependent (mono-cultures vs. co-cultures; human vs. mouse data).

It is also noteworthy that eCB-dysregulation may also contribute to the development of vitiligo, a chronic skin disease characterized by localized or generalized de-pigmentation, having a rather complex, but chiefly autoimmune pathogenesis [162,163]. Indeed, similar to AA, the 1858 C/T missense single nucleotide polymorphism of PTPN22 (R620W; rs2476601) was shown to be associated with a higher vitiligo risk [164,165,166,167]. Interestingly, however, this association seems to be ethnicity-dependent, since no such correlation was found in Turkish and Jordanian patients [168,169]. Although one should keep in mind that the actual levels of eCBs have never been investigated in lesional skin of vitiligo patients, and that PTPN22 has other, ECS-independent biological functions [153], immunosuppressive cannabinoid signaling might have therapeutic value in vitiligo. This bold hypothesis has to be tested in future targeted studies. Putative melanocyte-related translational potential of the cannabinoid signaling modulation is summarized in Table 3.

### 2.4. Epidermal Keratinocytes

#### 2.4.1. Proliferation and Differentiation

Several members of the ECS (AEA, 2-AG, CB_1_, CB_2_, NAPE-PLD, FAAH, multiple TRP channels, etc.) have been shown to be expressed on human epidermal keratinocytes [45,91,92,95,111,133], and the functional activity of the putative EMT was also demonstrated on these cells [170]. What’s more, one of the first pieces of morphological and biochemical evidence indicating that transport and hydrolysis of AEA are two spatially and functionally distinct processes was also provided in HaCaT keratinocytes [171].

Based on the available functional evidence, the homeostatic eCB-tone appears to play a role in regulating proliferation/differentiation balance, as well as pro-inflammatory mediator production and release by epidermal keratinocytes. Indeed, activation of CB_1_ by 1 μM AEA was shown to prevent differentiation induced by the combination of 12-O-tetradecanoylphorbol 13-acetate (a “general” PKC activator) and elevated [Ca^2+^]_EC_ in confluent 2D keratinocyte cultures, as revealed by abrogated cornified envelope formation [170]. Importantly, AEA was also able to prevent differentiation-induced up-regulation of several differentiation markers (keratin (K)-1, K10, involucrin and transglutaminase 5) by increasing DNA methylation, through a p38, and, to a lesser extent, an ERK1/2 MAPK-dependent pathway, again, in a CB_1_-dependent manner [172,173]. On the other hand, higher (3–30 μM) concentrations of AEA were found to suppress proliferation and to induce apoptosis of HaCaT and primary human epidermal keratinocytes in vitro, as well as in situ in full-thickness human skin organ culture (hSOC) via sequentially activating first CB_1_ and then indirectly TRPV1 [174]. Likewise, 24-h treatment of hSOC with 30 μM arachidonyl-2′-chloro- ethylamide (ACEA; CB_1_-specific agonist) suppressed proliferation (monitored by the ratio of Ki-67 positive nuclei), and this effect could be abrogated by the CB_1_-selective antagonist/inverse agonist AM251 (1 μM). Intriguingly, although the above ACEA treatment also decreased staining intensity of two proliferation-associated keratins (K6 and K16), this effect could not be prevented by the said CB_1_ blocker [175]. Finally, in a pilot hSOC experiment, 48-hr treatment with 1 μM ACEA down-regulated K1 and up-regulated K10 expression [176].

In line with the above observations, 0.5–1 μM CBD and CBG (but interestingly, not CBDV) also exerted differentiation-impairing effects in HaCaT keratinocytes (suppression of K1, K10, involucrin and transglutaminase 5 expression) via increasing DNA methylation by selectively enhancing DNA (cytosine-5)-methyltransferase 1 (DNMT1) expression. Although CBG was found to act in a CB_1_ and CB_2_ independent manner, quite surprisingly, CB_1_ antagonism could partially prevent the action of CBD [59]. The role of non-classical cannabinoid targets in mediating pCB actions was further confirmed in another model system, where 1–10 μM of THC, CBD, CBN, and CBG exerted anti-proliferative actions (72-h treatments) on HPV-16 E6/E7 transformed human keratinocytes (“CRL-2309 KERT”), in a TRPV1, CB_1_ and CB_2_ independent manner [122].

These observations, albeit being slightly nebulous, collectively support the concept that slight/moderate CB_1_ activation may operate as suppressor of the differentiation, whereas its activation by high concentrations of AEA or ACEA rather leads to anti-proliferative and pro-apoptotic events. However, certain pieces of evidence suggest that the role of CB_1_ might be even more complex, and context-dependent.

#### 2.4.2. Barrier Formation

Indeed, by assessing wild-type as well as CB_1_^−/−^ and CB_2_^−/−^ global KO mice, another team showed that absence of CB_1_ delayed, whereas lack of CB_2_ accelerated permeability barrier recovery after tape-stripping [177]. In line with these observations, lamellar body secretion as well as expression of certain late differentiation markers (filaggrin, loricrin, involucrin, as well as ratio of apoptotic cells) were increased in CB_2_^−/−^ mice and were decreased/abnormal in CB_1_^−/−^ animals, suggesting that differentiation of epidermal keratinocytes was indeed less efficient in the latter case [177]. In line with these data, both topically applied AEA and a synthetic CB_1_ agonist (α-oleoyl oleylamine serinol; α-OOS) were found to accelerate barrier recovery following tape-stripping in another study [178]. Although the apparent contradiction between these in vivo animal data and the aforementioned findings obtained in cultured keratinocytes as well as in ex vivo hSOCs has not been resolved yet, one can speculate that the difference most likely lies at the base of the CB_1_ expression in other cell types, and hence in disturbed intercellular communication. Alternatively, delayed barrier repair in CB_1_^−/−^ animals may be due to the elevated baseline secretion of thymic stromal lymphopoietin (TSLP) [179], a pro-inflammatory mediator driving T_h_2-type cutaneous inflammation in AD, since T_h_2 cytokines are known to impair the epidermal barrier [180,181]. Further experiments, ideally using keratinocyte-specific CB_1_ and CB_2_ KO mice, are now invited to dissect the exact role of CB_1_/CB_2_ and eCB signaling in keratinocyte differentiation.

#### 2.4.3. Keratin Disorders

Epidermolytic ichthyosis (EI), pachyonychia congenita (PC) and epidermolysis bullosa (EB) are rare genodermatoses caused by function-impairing mutations in different keratins (EI: K1 or K10; PC: K6, K16 or K17; EB: K5 or K14) [182]. Thus, pharmacologically induced down-regulation of the mutated, dysfunctional keratins, and ideally, up-regulation of other ones capable of compensating the role of the mutated molecules, is thought to be an innovative, novel approach in these diseases [176,182]. Since irrespective of the above open questions, it seems to be safe to assume that appropriate modulation of the eCB signaling and/or administration of various pCBs may be capable of inducing marked alterations in the keratin expression profile in human epidermis, it is not surprising that such interventions were already suggested to be exploited in these diseases [176,182].

Along these lines, it is important to note that according to a recent observational study reporting 3 cases of self-initiated topical CBD use in patients with EB, CBD may improve quality of life in such patients. Indeed, one patient was weaned completely off oral opioid analgesics, and all 3 patients reported faster wound healing, less blistering, and amelioration of pain. The authors concluded that the effects might have been due to the anti-inflammatory activity of CBD, but in light of the above data, one can speculate that CBD might have beneficially modulated the keratin expression profile as well [183]. Likewise, in another small pilot study, three EB patients, who were prescribed pharmaceutical-grade sublingually administered cannabinoid-based medicine (CBM) comprising THC and CBD, reported improved pain scores, reduced pruritus and decreased overall analgesic drug intake [184]. Further studies are therefore invited to exploit putative therapeutic potential of the (endo)cannabinoid signaling in the clinical management of keratin diseases.

Putative keratinocyte-related translational potential of cannabinoid signaling modulation is summarized in Table 4.

### 2.5. Cutaneous Inflammation

#### 2.5.1. General Considerations

Another key function of cannabinoid signaling is to control local immune responses in the skin. Several lines of evidence demonstrate that both eCBs and pCBs can modulate immune functions, and they are generally considered to be anti-inflammatory agents [31,33,141,142]. Of great importance, immune effects of cannabinoids are not only exerted on “professional” immune cells, but also on non-immune cells (e.g., keratinocytes, sebocytes).

As mentioned above, many cell types of the skin express pathogen- and danger-associated molecular pattern recognizing receptors. These cells are also capable of producing anti-microbial peptides and lipids, and can initiate and coordinate local immune responses as well, by producing various pro- and anti-inflammatory cytokines and chemokines [4,16,17,18,20]. These processes are under the tight control of the cutaneous cannabinoid system [33,91,92,93].

Indeed, as it was elegantly demonstrated in the groundbreaking work of Karsak and her co-workers, homeostatic eCB-signaling through CB_1_ and CB_2_ receptors is a key mechanism, which keeps the production and release of pro-inflammatory cytokines and chemokines under control in epidermal keratinocytes [143]. Dinitrofluorobenzene (DNFB)-induced allergic inflammation was more severe in CB_1_^−/−^/CB_2_^−/−^ double KO mice as compared to the wild-type, whereas the inflammatory response was significantly suppressed in FAAH^−/−^ animals, as well as in THC-treated (5 mg/kg subcutaneously injected or 30 μg topically administered) wild-type mice. Intriguingly, however, the CB_2_-selective agonist HU-308 (5 mg/kg subcutaneously injected or 10 μg topically administered) failed to induce significant alleviation, suggesting that both CB_1_ and CB_2_ are needed for the effect in this inflammatory model system [143].

In line with this concept, 24-h treatment with the TLR4 activator LPS (5 μg/mL) was found to up-regulate CB_1_ and CB_2_ mRNA expression in primary human keratinocytes. Moreover, in the presence of 10 μg/mL LPS, the CB_2_-selective JWH-015 promoted wound closure (scratch assay of human keratinocyte-fibroblast co-culture), elevated TGF-β-release, and exerted anti-inflammatory effects in a CB_1_ and CB_2_-dependent manner. Since JWH-015 could be successfully delivered into porcine skin, the authors concluded that it may be a powerful future anti-inflammatory agent [185]. Similarly, novel synthetic CB_2_-activators suppressed chemokine (C-C motif) ligand 8 (CCL8; a.k.a. monocyte chemoattractant protein 2 [MCP-2]) release from poly-(I:C)-stimulated (100 μg/mL; 6 h) HaCaT keratinocytes in a CB_2_-dependent manner, since co-administration of AM630 (100 nM) could prevent the action [186].

The fundamental role of homeostatic eCB-signaling in controlling epidermal inflammatory responses was further supported by a recent study demonstrating that activation of TLR2 by lipoteichoic acid (LTA; 10 μg/mL; 24 h) led to the up-regulation of FAAH-activity as well as expression at the protein (but intriguingly, not at the mRNA) level in human keratinocytes [144]. Moreover, FAAH-inhibitors could prevent the LTA-induced pro-inflammatory response in a CB_1_/CB_2_ receptor-dependent manner. Co-administration of the CB_1_ and CB_2_ antagonists/inverse agonists AM251 and AM630 (both at 1 μM) prevented the action; however, the compounds were only tested in combination, leaving the individual roles of CB_1_ and CB_2_ unexplored. Moreover, following topical application, the FAAH-inhibitors alleviated dust mite-induced cutaneous inflammation of NC/Tnd mice with the same efficiency as the positive control tacrolimus [144]. Likewise, topical administration of sulfur mustard and nitrogen mustard at concentrations that induced tissue injury in mice led to up-regulation of FAAH (as well as of CB_1_, CB_2_, and PPARα). These alterations persisted throughout the wound healing process, and FAAH-inhibitors were found to be highly effective in suppressing vesicant-induced cutaneous inflammation in this study too [187]. Collectively, these data highlight the possibility that by regulating homeostatic eCB signaling, FAAH may be an important regulator of the initiation and maintenance of cutaneous inflammatory processes. Thus, restoration of the homeostatic eCB tone by e.g., FAAH-inhibitors may be a promising tool in alleviating skin inflammation [93].

Besides eCBs and THC, other pCBs also deserve attention as potential topical anti-inflammatory agents. Indeed, in a croton oil-induced murine cutaneous inflammation model [188], topical administration of several pCBs (CBC, CBCV, CBD, CBDV, Δ^8^-THCV, Δ^8^-THC, Δ^9^-THC; 0.1–1 μmol/cm^2^) was found to exert significant anti-inflammatory effects as revealed by reduced ear swelling [189]. Moreover, in poly-(I:C)-stimulated HaCaT cells (100 μg/mL, 6 h), CBD (5–20 μM) elevated the levels of AEA and concentration-dependently inhibited poly-(I:C)-induced release of CCL8 (a.k.a. MCP-2), IL-6, IL-8, and TNF-α. The effects could be reversed by CB_2_ (AM630; 0.1 μM) and TRPV1 (5’-iodo-resiniferatoxin [I-RTX]; 1 μM) antagonists, without any cytotoxic effect. Importantly, low micromolar (1–20 μM) concentrations of THCV, CBC and CBG were also efficient, but exhibited inferior efficacy compared to CBD [190]. Finally, as mentioned above, CBD (10 μM; A_2A_ receptor dependent action) [120], as well as CBG, CBGV, CBC, CBDV, and THCV (all in 0.1 μM) [126] were found to exert anti-inflammatory effects in human sebocytes, whereas CBD (0.1 μM; adenosine receptor dependent action) was also shown to be effective in alleviating poly-(I:C)- induced pro-inflammatory response in cultured human plucked HF-derived outer root sheath (ORS) keratinocytes [140].

Interestingly, other in vitro and in vivo studies have found that in certain inflammation models activation of CB_1_ alone may also be sufficient to induce potent anti-inflammatory actions. Indeed, the IFN-γ-induced pro-inflammatory response (elevated production of T_h_1- and T_h_17-polarizing cytokines IL-12 and IL-23) was prevented by 2.5 μM AEA pre-treatment in HaCaT keratinocytes in a CB_1_-dependent manner [191]. Moreover, keratinocyte-specific CB_1_^−/−^ mice exhibited a stronger pro-inflammatory reaction (higher up-regulation of IL-4, CCL8 [a.k.a. MCP-2], TSLP, and eosinophilic activity) in fluorescein isothiocyanate (FITC)-induced atopic-like inflammation, and showed delayed barrier repair following FITC challenge. Furthermore, keratinocytes of keratinocyte-specific CB_1_^−/−^ mice secreted more TSLP under un-stimulated conditions [179]. By using the same mice strain, very similar data (increased and prolonged contact hypersensitivity responses with enhanced reactive epidermal acanthosis and inflammatory keratinocyte hyperproliferation) were obtained in DNFB-induced cutaneous inflammation. Finally, primary cultures of CB_1_-deficient keratinocytes released increased amounts of CXCL10 and CCL8 (a.k.a. MCP-2) after stimulation with IFN-γ, highlighting keratinocyte CB_1_-signaling as a master regulator of T cell-dependent cutaneous inflammation in the effector phase of contact hypersensitivity [192].

Surprisingly, however, certain experimental data appear to contradict this simplistic picture. Indeed, 4 kJ/m^2^ (400 mJ/cm^2^) UVB-irradiation induced inflammation in wild-type mice; however, CB_1_^−/−^/CB_2_^−/−^ double KO animals appeared to be protected [87]. Moreover, the same UVB irradiation was shown to induce fast (≤30 min) phosphorylation and internalization of CB_1_ and CB_2_ in overexpressor HEK293 cells, and it also activated ERK1/2, p38 and JNK MAPK cascades in wild-type, but not in CB_1_^−/−^/CB_2_^−/−^ double KO, mouse embryonic fibroblasts [87]. Finally, elevation of TNF-α level following UVB treatment was higher in the epidermis of wild-type than in the epidermis of CB_1_^−/−^/CB_2_^−/−^ double KO mice [87]. Since the authors found that UVB (9 kJ/m^2^→900 mJ/cm^2^) or UVA (60 or 120 kJ/m^2^→6 and 12 J/cm^2^) irradiation induced a substantial lowering in K_i_ values in a competition binding assay using membrane fractions of CB_1_ or CB_2_ overexpressing cells, they concluded that CB_1_ and CB_2_ could directly be activated by UV-irradiation. Thus, the UVB→CB_1_/CB_2_→NF-κB activation axis was suggested to play a key role in UV-induced inflammation [87].

At this point, it is important to note that physiological relevance of such high UV doses is questionable, since the minimal erythema dose of narrow-band UVB irradiation phototype-dependently ranges typically between ~300 and 900 mJ/cm^2^. However, the observed phenomena may contribute to the beneficial therapeutic effects in psoriasis and scleroderma, since the maximal doses of UVB and UVA for psoriasis or scleroderma treatment may reach 1.5 J/cm^2^ (UVB, psoriasis) and 130 J/cm^2^ (UVA, scleroderma) [193,194,195].

Finally, to add a further layer to the complexity of the system, it is noteworthy that another cannabinoid-responsive receptor (namely TRPV4) was also found to play a role in detecting UVB. Indeed, UVB-induced sunburn and pain was found to be mediated via direct (i.e., UVB-induced) activation of TRPV4 ion channels in epidermal keratinocytes, and the subsequent release of endothelin-1 [86].

#### 2.5.2. Role of “Non-Classical” Cannabinoid Targets

Having discussed the importance of keratinocyte CB_1_ (and CB_2_) mediated (mostly) anti-inflammatory signaling, it should also be noted that several lines of evidence highlight the existence of additional, so far un-identified, non-classical anti-inflammatory cannabinoid pathways in the skin. Indeed, topical application of THC (30 μg) was found to be efficient in alleviating DNFB-induced allergic ear swelling and myeloid immune cell infiltration not only in wild-type but also in CB_1_^−/−^/CB_2_^−/−^ double KO mice. Moreover, THC suppressed the IFN-γ production of CD3+ T cells, decreased the release of CCL2 and of IFN-γ-induced CCL8 and CXL10 from epidermal keratinocytes, and limited the recruitment of myeloid immune cells in vitro in a CB_1_/CB_2_ receptor-independent manner [196].

Obviously, in case of pCBs, potential effects (activation, antagonism or desensitization) on various TRP channels and many other targets (e.g., adenosine receptors or PPARs) have to be taken into consideration [31,32,33,35,40,41,45,46,53,54,55,56,57] as well; thus, their “net” biological effects will always be determined by a mixture of multiple molecular actions. With respect to this point, albeit detailed overview of the roles of TRP channels, adenosine receptors, and PPARs in cutaneous biology lies far beyond the scope of the current review, we have to emphasize that the activation of the most skin-relevant TRP channel, i.e., TRPV3 [197], results in an elevated production and release of several pro-inflammatory cytokines from human epidermal keratinocytes [121] and human sebocytes [119]. Thus, the ability of CBD, THCV and CBGV to activate (and then desensitize) TRPV3 [55] may also contribute to their context-dependent pro- or anti-inflammatory actions. Moreover, considering the concentrations needed to activate anti-inflammatory adenosine receptors (high nanomolar range in case of CBD in plucked HF-derived outer root sheath keratinocytes [140] or 1 μM in murine brain “b.end5” endothelial cells [198]) and the rather pro-inflammatory TRPV channels (low micromolar range [55,56]), their efficiency may theoretically exhibit reverse dose-dependence, i.e., superior anti-inflammatory activity at the more adenosine receptor-specific nanomolar than in the TRPV-activating micromolar concentrations.

#### 2.5.3. Role of “Non-Classical” Cannabinoid Ligands

Besides the “classical” pCBs, plant-derived active substances exhibiting potential cannabimimetic effects have also been investigated in various model systems. Indeed, CB_2_ activating *Echinacea purpurea*-derived alkylamides were shown to reduce the TLR3 activator poly-(I:C) (20 μg/mL; 3 h) induced mRNA expression as well as release of pro-inflammatory cytokines (IL-6 and IL-8) in HaCaT keratinocytes; however, it has not been investigated whether the actual effects were indeed coupled to the activation of CB_2_. The same *Echinacea* extract containing Linola^®^ Plus Cream was proven to be well-tolerated, and it reduced local SCORAD not only compared to baseline, but also compared to a comparator product Imlan^®^ Creme Pur. Moreover, it resulted in significantly improved lipid barrier (with higher levels of overall epidermal lipids, ceramide EOS [ω-esterified fatty acid+sphingosine sphingoid base], and cholesterol at day 15 compared to baseline as well as significantly greater number of intercellular lipid lamellae) in respective clinical trials [199].

With respect to the non-classical ECS-related endogenous ligands, it is noteworthy that orally administered (10–30 mg/kg) PEA exerted sustained anti-inflammatory effects in spontaneously *Ascaris* hypersensitive Beagle dogs, which were challenged with intradermal injections of *Ascaris suum* extract, substance P, and anti-canine IgE [200]. Moreover, in HaCaT cells, stimulation with poly-(I:C) (100 μg/mL; 24 h) elevated the levels of PEA, OEA, and AEA (but decreased the level of 2-AG). Moreover, exogenous PEA (10 μM) inhibited poly-(I:C)-induced expression and release of CCL8 (a.k.a. MCP-2), in a TRPV1- (but not PPARα) dependent manner [200].

Intraperitoneally applied PEA (5–10 mg/kg) was also able to inhibit DNFB-induced ear inflammation in mice in vivo, in a TRPV1-dependent manner. Moreover, DNFB treatment increased ear skin PEA levels (interestingly, in CB_1_/CB_2_ double KO mice, the elevation was higher than in wild-type), and up-regulated TRPV1, PPARα and NAPE-PLD (PEA and AEA synthesizing enzyme) in keratinocytes [201]. Importantly, the authors reported that PEA (5 mg/kg; i.p.) reduced ear swelling, the number of mast cells, as well as the expression of VEGF and its receptor FLK-1 in a CB_2_-dependent, but PPARα-independent manner in the late, allergic stage of the same model system, whereas the anti-pruritic effect of PEA was mediated in a CB_2_- and PPARα-dependent manner [202]. Interestingly, PEA and OEA, but not AEA or 2-AG, were up-regulated in the epidermis of sodium lauryl sulfate (SLS)-challenged (2.5%; 24 h) buttock skin of 10 healthy volunteers. Although UVB-irradiation, which resulted in a similar erythema, had no effect on the above eCB levels [203], UVA- and UVB-irradiation of human CDD 1102 KERTr keratinocytes (UVA: 30 J/cm^2^; UVB: 60 mJ/cm^2^) and CCD 1112Sk fibroblasts (UVA: 20 J/cm^2^; UVB: 200 mJ/cm^2^) decreased cytosolic, and increased cell membrane CB_1_, CB_2_ and TRPV1 expression (post-irradiation day 1). Intriguingly, both UVA and UVB irradiation were found to decrease AEA levels, whereas 2-AG was only reduced by UVB [204]. Although the authors did not investigate if the “cytosolic” CB_1_ fraction represents mitochondrial [81,82] or lysosomal [83] CB_1_ expression, one might speculate that the elevated production of reactive oxygen species (ROS) observed upon UV-irradiation might have been (at least in part) due to a reduced mitochondrial CB_1_ expression leading to increased mitochondrial activity. However, putative expression and functional role of intracellular CB_1_ sub-populations in epidermal keratinocytes remains to be elucidated in future targeted studies.

Finally, with respect to PEA it should also be noted that a PEA- and organic osmolyte-containing topical product (Physiogel^®^ A.I. Cream) significantly inhibited the development of UV light (UVB 20%, UVA 80%; produced by a solar UV simulator)-induced erythema and thymine dimer formation in normal human skin. However, it did not alter the ratio of Ki-67+ proliferating keratinocytes and the expression of p53 and ICAM-1. Hence, PEA might become a novel tool to alleviate UV-induced photodamage [205].

#### 2.5.4. Putative ECS- Endogenous Opioid System (EOS) Interplay

As mentioned above, the ECS may interact with several other signaling pathways, including the endogenous opioid system (EOS). Indeed, intraplantar administration of the CB_2_-selective agonist AM1241 (10 μM) stimulated β-endorphin release from keratinocytes via the activation of a CB_2_-G_i/o_-G_βγ_-ERK1/2 MAPK-Ca^2+^ signaling pathway [206]. The released β-endorphin was then found to activate local neuronal μ-opioid receptors thereby inhibiting nociception in rats, which was not the case for CB_2_^−/−^ animals [207]. Similarly, capsaicin-induced pain was dose-dependently alleviated in mice by intraplantar injection of the highly CB_2_-selective agonist β-caryophyllene (18 μg) [208], most likely via stimulating β-endorphin release from the keratinocytes.

Intriguingly, further ECS-EOS interplay was evidenced in a few additional studies. Indeed, electroacupuncture (EA) was found to increase CB_2_ expression on keratinocytes and infiltrating inflammatory cells in inflamed skin tissues of rats [209]. EA and CB_2_ stimulation reduced inflammatory pain via activating μ-opioid receptors, and EA increased endogenous opioid expression in keratinocytes as well as in infiltrating immune cells at the inflammatory site through CB_2_ activation [210]. Furthermore, EA or AM1241 (1 mg/kg; s.c.) treatment significantly decreased the mRNA and protein levels of IL-1β, IL-6 and TNF-α in inflamed skin tissues in a CB_2_-dependent manner, since pretreatment with the CB_2_-selective antagonist/inverse agonist AM630 (150 μg/kg; s.c.) abrogated the effect of EA. Collectively, these data suggest that EA may reduce inflammatory pain and pro-inflammatory cytokine production by activating CB_2_ [211].

#### 2.5.5. Selected “Skin-Relevant” Professional Immune Cells: Langerhans Cells and Mast Cells (MC)

As we discussed above, several lines of evidence demonstrate that cutaneous cannabinoid signaling profoundly influences the immunogenic behavior of skin resident non-immune cells. Unfortunately, albeit effects of cannabinoid signaling on immune cells in general are well documented [31,33,141,142,212,213], much less data are available about their skin-relevant aspects.

Indeed, according to the sole available paper, murine epidermal Langerhans cells express CB_2_ at the mRNA level. Moreover, the authors showed that 2-AG level was increased in oxazolone-induced dermatitis, and that treatment with the CB_2_-selective antagonist SR144528 attenuated the inflammatory response; thus, they concluded that “CB_2_ and 2-AG play important stimulative roles in the sensitization, elicitation, and exacerbation of allergic inflammation” [214]. Although it cannot be excluded that 2-AG→CB_2_ signaling axis may model- and context-dependently play such roles, the data should be interpreted carefully, since the authors could not find CB_2_ positivity in epidermal keratinocytes. Thus, targeted studies are urgently invited to explore ECS of Langerhans cells, preferably in human skin, or in human monocyte-derived model systems.

In contrast to Langerhans cells, several cannabinoid-relevant studies have been conducted on different mast cell (MC) models, among which, we summarize the most important and skin-relevant ones below. MCs are important professional immune cells of the cutaneous immune system. They are able to detect several different potential danger signals, and, by producing and on-demand releasing a number of different soluble mediators, they can influence a wide-array of biological processes, including tissue remodeling, wound healing, fibrosis, local immune responses, itch, or even hair growth [215,216,217,218,219,220,221,222,223,224,225]. Although there are a number of cell lines (rat: RBL-2H3; human: HMC-1, LAD1, LAD2, etc.) generally capable of mimicking several aspects of human MC biology, one should not forget how important environmental signals are in regulating and fine-tuning MC activity [215,216,217,218,219,220,221,222,223,224]. Maybe because of this limitation of the in vitro systems, partially conflicting results have been obtained with respect to the effects of the cannabinoid signaling.

First, PEA was identified as an endogenous activator of CB_2_ on RBL-2H3 cells as well as Wistar rat peritoneal MCs, where its administration resulted in an anti-inflammatory phenotype, whereas AEA was found to be ineffective [226]. Later, PEA (1-10 μM) was shown to suppress anti-canine IgE-induced activation of skin MCs ex vivo in freshly isolated dog skin specimens [227], while in another study, enhanced local MC proliferation and (maybe compensatory) elevation of levels of PEA and other bioactive lipid mediators were found in canine AD [228]. Finally, the NAAA-inhibitor 2-pentadecyl-2-oxazoline-derivative of PEA (“PEA-OXA” 10 mg/kg p.o.) reduced MC activation in carragenan–induced inflammation in rats in a PPARα-independent manner [229]. Last, but not least, ultramicronized PEA (PEA-um) decreased compound 48/80-induced vasodilation and MC degranulation in organ-cultured skin of dogs [230]. Taken together, the available evidence strongly suggests that appropriately chosen concentrations of PEA may be efficient in suppressing MC degranulation in the skin [231].

Next, by using RBL-2H3 cells and bone marrow MCs, another group found that 1–10 μM of metAEA (a FAAH-resistant AEA-analogue) increased the level of cAMP (2 h), and suppressed anti-DNP IgE-induced degranulation in a CB_1_-dependent manner, whereas the CB_2_-selective agonist JWH-015 decreased cAMP level in a CB_2_-dependent manner [232]. Interestingly, CBD (3–10 μM) and THC (15 μM) were found to trigger activation of RBL-2H3 cells via inducing Ca^2+^-influx. Although the mechanism of action was not uncovered in this study, one might speculate that CBD and THC might have activated certain TRPV channels, which were already shown to mediate MC activating signals [233,234,235].

On the other hand, WIN55,212-2 and CP 55,940 (two non-selective synthetic cannabinoids activating both CB_1_ and CB_2_) could prevent IgE-DNP-induced activation of RBL-2H3 cells [236]. Finally, semi-synthetic CB_1_ activators as well as AEA (10 μM) inhibited the release of inflammatory mediators without causing cytotoxicity in RBL-2H3 cells, and dose-dependently suppressed MC proliferation. Topical application of the above CB_1_ agonists suppressed the recruitment of MCs into the skin in an oxazolone-induced mouse model of AD, and reduced the blood level of histamine [237].

By using the human HMC-1 cell line, another group described functionally active EMT and inducible FAAH expression in MCs, but they did not find CB_1_ or CB_2_ expression [238], in spite of the fact that presence of CB_1_ and CB_2_ was shown in human skin MCs [111]. Moreover, in HMC-1 cells neither AEA nor PEA (10 μM both) affected tryptase release triggered by 500 ng/mL A23187 (a Ca^2+^ ionophore) [238]. Interestingly, unlike CB_1_ and CB_2_, GPR55 was found to be expressed on HMC-1 cells. In this study, PEA was found to reduce PMA (a general activator of classical and novel PKC isoforms) induced nerve growth factor (NGF) release in a GPR55-dependent manner (confirmed by GPR55 RNA_i_). Thus, by regulating NGF release from activated MCs, PEA was suggested to influence NGF-induced angiogenesis [239].

In contrast to the above data, 30 μM WIN55,212-2 was found to CB_2_-dependently prevent degranulation of LAD2 cells induced by the supernatant of human HPV18-positive SW756 cervical carcinoma cells [240]. Moreover, AEA inhibited FcεRI-dependent degranulation and cytokine synthesis in murine bone marrow-derived MCs via the activation of CB_2_/GPR55 receptor heteromers [241], and VCE-004.3, as well as VCE-004.8, two PPARγ and CB_2_ receptor activating derivatives of CBD, could also reduce MC degranulation in bleomycin-induced murine fibrosis [242,243].

It is also noteworthy that over activation of the aforementioned pro-inflammatory [119,121], and skin-wise highly relevant [197] TRPV3 ion channel may promote MC proliferation too. Indeed, DS-*Nh* mice and WBN/Kob-*Ht* rats (possessing Gly573 to Ser [“*Nh*” mutation] or Gly573 to Cys [“*Ht*” mutation] gain-of-function mutations of TRPV3) exhibiting hairless phenotype and suffering from pruritic dermatitis, were reported to have increased MC numbers. This supported the concept that TRPV3 might promote MC proliferation and activity [244], inviting the hypothesis that appropriate doses of TRPV3-desensitizing pCBs might exert MC-suppressive effects too.

As discussed above, the available cellular model systems provided somewhat controversial data especially with respect to the expression and role of CB_1_ and CB_2_, which might have been the consequence of the lack of appropriate tissue microenvironment. To overcome these issues, unconventional methods to study human MC biology were also employed. By using human HF, as well as human nasal polyp organ cultures to study the biology of MCs *in situ*, a crucial regulatory role for CB_1_ was demonstrated [245,246]. Although expression of CB_2_ was not confirmed [245], both HF connective tissue sheath and mucosal MCs were shown to be tightly controlled by the ECS. Indeed, excessive activation and maturation of MCs from resident progenitors was limited via tonic CB_1_ stimulation by locally synthesized eCBs [245,246]. Thus homeostatic eCB signaling, and especially appropriate function of CB_1_ appears to be a key gate-keeper of MC functions in situ, therefore elevation of the eCB-tone, administration of PEA as well as blockade/desensitization of certain TRP channels by well-selected doses of certain pCBs hold out the promise of having great translational potential as potent suppressors of unwanted MC overactivation.

#### 2.5.6. Selected Inflammatory Diseases: Psoriasis (PSO)

Psoriasis (PSO) is a chronic inflammatory skin disorder, often accompanied by additional non-cutaneous symptoms (e.g., arthritis), and its pathogenesis is still not fully understood. Indeed, genetic [247] and epigenetic [248] abnormalities, as well as alterations in the cutaneous microbiota [249], pH [250], or, most importantly, IL-17 signaling [251,252] are known to be involved in its development, and it is surely accompanied by a disturbance in the dynamic cross-talk between epidermal keratinocytes and professional cutaneous immune cells. This inappropriate communication then leads to pathological inflammatory processes and to a disturbance in the proliferation/differentiation balance of epidermal keratinocytes [249,253,254,255,256]. Since, as discussed above, proliferation/differentiation as well as immune activity of epidermal keratinocytes are under the tight control of the eCB signaling, it is not surprising that therapeutic exploitation of various cannabinoids in PSO has already been suggested by multiple authors [237,257,258,259,260,261].

Beyond of the abovementioned theoretical reasons (i.e., dose-dependent differentiation- modulating, as well as anti-proliferative and anti-inflammatory effects of various cannabinoids in the skin), there are a few additional pieces of evidence supporting the concept that eCB-dysregulation may contribute to the development of PSO. Indeed, the promoter of the PTPN22 gene was found to be hypomethylated resulting in its strong up-regulation in lesional skin of PSO patients as compared to the adjacent non-lesional skin [262]. Intriguingly, however, the C1858T substitution (“R620W variant”; “rs2476601”; a loss-of-function single-nucleotide polymorphism) in PTPN22 was found to be positively associated with PSO in Saudi patients [263], and other SNPs (“rs3789604”, “rs1217414”, “rs6679677”) were also found to be related to PSO in other subjects [264,265,266]. Others, however, found that C1858T substitution is only associated with higher susceptibility of psoriatic arthritis, but not of PSO itself [267,268,269], whereas again others did not find any significant association between PTPN22 and PSO [270,271,272,273,274], leaving the putative role of PTPN22 dysfunction in PSO rather controversial.

A much more important indicator of the potential involvement of eCB dysregulation in the pathogenesis of PSO is that a recent study found elevated AEA and 2-AG levels in the plasma of these patients. Moreover, in the granulocytes of the patients, activities of FAAH and MAGL were increased, and GPR55 expression was also up-regulated. With respect to the “classical” receptors, the authors found that expression CB_1_ was only increased in granulocytes of patients suffering from psoriatic arthritis, whereas CB_2_ was up-regulated in those PSO patients, who had no joint complications [275]. Moreover, RNAseq of skin biopsies obtained from 25 PSO patients revealed that, compared to region-matched skin of healthy subjects, several important “cannabinoid- relevant” genes were differentially expressed. Findings in this study include, but are not limited to down-regulation of adenosine A_1_, A_2A_, A_2B_ and A_3_ receptors, CB_1_, CB_2_, PPARα and PPARγ, whereas FAAH1 (but not FAAH2), TRIB3, TRPV1 and TRPV3 were up-regulated at the mRNA level in itchy lesional skin of PSO patients [276]. Thus, alterations in the ECS can indeed be observed in PSO patients, indicating that certain cannabinoids may possess therapeutic potential.

Along this line, it is important to emphasize that NRIP1, which has previously been shown to be an important CBD target gene [120], was found to be overexpressed both in skin and peripheral blood monomorphonuclear cells (PBMC) of PSO patients [277]. Importantly, its down-regulation in HaCaT keratinocytes could significantly suppress proliferation and induce apoptosis, whereas in isolated CD4+ T cells it reduced RelA/p65 NF-κB expression and IL-17 release [277]. Moreover, in NRIP1^−/−^ mice, the PSO-mimicking inflammation induced by imiquimod (a TLR7/8 agonist widely used to trigger PSO-like cutaneous symptoms in mice [278]) was delayed, and RelA/p65 NF-κB expression was also reduced in the lesions [277]. Collectively, these data suggested that NRIP1 may be a multifaceted therapeutic target in PSO. Since CBD was found to TRPV4-dependently down-regulate NRIP1 in human sebocytes [120], one might speculate that, by activating the same signaling axis, it could exert beneficial effects in PSO as well. On the other hand, it is also important to note that another CBD target gene, namely TRIB3, which was shown to be adenosine A_2A_ receptor-dependently up-regulated in human sebocytes [120], was found to be up-regulated in PSO lesions compared to non-lesional skin, and TRIB3-silencing exerted anti-proliferative effects in HaCaT keratinocytes [279]. Further studies are therefore invited to explore how CBD regulates these PSO-relevant signaling pathways in actual patients.

#### 2.5.7. Selected Inflammatory Diseases: AD

Although AD and PSO are two markedly different diseases, their pathogeneses still show some similarities in certain aspects. Indeed, impaired keratinocyte differentiation leading to defects in the cutaneous barrier functions, as well as disturbed keratinocyte—immune cell communication and pathological inflammatory processes can be observed in both diseases, but, obviously, the exact contributors (i.e., involved key cytokines, dysregulated barrier genes, etc.) are different [280,281,282,283,284,285,286,287]. Thus, similar to PSO, cannabinoid signaling may theoretically possess therapeutic value in AD as well [288,289,290].

Indeed, in skin samples of dogs suffering from AD, CB_1_ and CB_2_ immunoreactivity [291], as well as levels of PEA [228] were shown to be higher than in skin samples of healthy animals, and not less than 18 genetic variants of PTPN22 were shown to be likely to be associated with AD in West Highland white terriers [292]. With respect to the human data, it is noteworthy that RNAseq of skin biopsies obtained from 25 AD patients revealed that, compared to region-matched skin of healthy subjects, several important “cannabinoid-relevant” genes were differentially expressed. These included, but were not limited to the finding that CB_1_, CB_2_ and GPR18 were down-regulated, whereas TRPV1 and TRPV2 were up-regulated at the mRNA level in itchy lesional skin of AD patients [276]. Thus, alterations in the ECS can indeed be observed in AD, indicating that certain cannabinoids may possess therapeutic potential.

Indeed, pharmacological blockade of TRPV1 has recently emerged as potential novel therapeutic possibility in managing AD [293], and according to certain in vitro and animal data, TRPV3 antagonists also seem to be promising anti-AD candidate drugs [119,121,244]. Future studies are therefore urgently invited to explore if TRPV3 desensitizing pCBs [55], most especially CBGV, which has been proven to exhibit anti-inflammatory and moderate sebostimulatory effects [126], indeed exert beneficial effects in AD.

With respect to the “classical” receptors, it is noteworthy that the orally available CB_2_ agonist S-777469 (1–10 mg/kg) significantly suppressed DNFB-induced ear swelling in BALB/c mice in a dose-dependent manner, and alleviated mite antigen-induced AD-like skin lesions in NC/Nga mice (10–30 mg/kg) as revealed by reduced epidermal thickness, as well as MC and eosinophil numbers. Moreover, dust mite-challenge was found to elevate the 2-AG level in the skin of NC/Nga mice, while S-777469 could suppress 2-AG (0.5 μM)-induced migratory response of differentiated EoL-1 (human eosinophilic leukemia cell line) and HL-60 (human monocytic cell line) cells in vitro. Thus, the authors concluded that S-777469 may act via inhibiting cutaneous inflammation by blocking the actions of 2-AG [294].

Although the concept that CB_2_ activation may be beneficial in AD was further supported by a recent study demonstrating the efficiency of a CB_2_-activating *Echinacea purpurea* extract in alleviating AD symptoms [199], a few additional data argue that the overall picture may be more complex. JTE-907, a CB_2_ antagonist/inverse agonist, was found to exert anti-pruritic activity in NC mice suffering from chronic AD-like dermatitis [295]. In line with these observations, in another study JTE-907 as well as SR144528 (another CB_2_ blocker) suppressed DNFB-induced ear swelling (0.1–10 mg/kg p.o. in both cases), probably via inhibiting 2-AG→CB_2_-driven migration of certain immune cells [296].

Intriguingly, unlike CB_2_, CB_1_ was found to exert clearly beneficial effects in murine cutaneous inflammation models. Indeed, as mentioned above, topical application of AEA (0.5%) as well as of α-oleoyl oleylamine serinol (α-OOS; a newly developed CB_1_ agonist; 1%) were shown to accelerate epidermal permeability barrier recovery following tape-stripping, as revealed by transepidermal water loss measurement [178,237], whereas lack of CB_1_ was found to delay epidermal barrier recovery in CB_1_^−/−^ mice [177]. Moreover, administration of α-OOS resulted in anti-inflammatory effects in both acute (12-O-tetradecanoylphorbol-13-acetate-induced) and chronic (oxazolone- induced) inflammation models [178,237]. Further details of the potent cutaneous anti-inflammatory effects of CB_1_ are reviewed above (see Section 2.5.1). Finally, highly selective FAAH-inhibitors (WOBE440 and -479) could efficiently alleviate dust mite-induced “atopic-like” cutaneous inflammation in NC/Tnd mice [144]. 

With respect to the “non-classical” cannabinoids, it is noteworthy that the NAAA-inhibitor ARN077 dose-dependently suppressed edema formation and scratching in DNFB-induced dermatitis. Moreover, it also increased tissue PEA content, and normalized circulating levels of various cytokines (IL-4, IL-5, IFN-γ) and IgE in a PPARα-dependent manner, since the effects did not develop in PPARα^−/−^ mice. Thus, NAAA-inhibition and the elevation of PEA level were identified as a promising tool in AD and maybe in other inflammatory disorders of the skin [297].

In another study, PEA was found to selectively activate PPARα in vitro (EC_50_ = 3.1 ± 0.4 μM), and it up-regulated mRNA expression of PPARα following topical application to mouse skin. Moreover, in carrageenan-induced paw edema as well as in phorbol ester-induced ear edema, PEA was found to attenuate inflammation in wild-type mice, but had no effects in PPARα^−/−^ animals. Importantly synthetic PPARα agonists GW7647 (150 nmol/cm^2^ topically) and Wy-14643 (20 mg/kg; i.p.) PPARα-dependently mimicked these effects, and the edema suppressing activity of OEA was also mediated by PPARα [298]. In line with these observations, PEA-um was found to be effective and safe in reducing pruritus and skin lesions, as well as in improving quality of life in dogs with moderate AD and moderate pruritus [299]. Last, but not least, the “ATOPA” study assessing efficiency of a special PEA-containing cream (Physiogel^®^ A.I. Cream) found a substantial improvement in the objective and subjective symptoms (decline of pruritus and loss of sleep) of AD after regular skin care with the cream, and a reduced use of topical corticosteroids was also observed [300]. In line with these observations, PEA and N-acetylethanolamine were found to be effective in asteatotic AD in a randomized, double-blind, controlled study involving 60 patients [301].

#### 2.5.8. Selected Inflammatory Diseases: Systemic Sclerosis (SSc)

Systemic sclerosis (SSc) is a chronic autoimmune disease characterized by vascular abnormalities, and fibrosis of the skin and of other organs, including the heart, kidneys, lungs, etc. Its etiology is still nebulous, but genetic [302] and epigenetic [303,304] factors, as well as abnormalities in the gut microbiota [305], and oxidative stress [306] were shown to play a role in its development. The initial trigger is considered to be an autoimmune reaction against endothelial cells leading to the characteristic vascular abnormalities, but inappropriate immune cell—fibroblast cross-talk leading to progressive fibrosis and differentiation of fibroblasts to α-smooth muscle actin (α-SMA) positive myofibroblasts are also very important [307].

Similar to many other diseases with an autoimmune component, the association between PTPN22 SNPs and SSc was already suggested by multiple studies [308]. Indeed, the aforementioned R620W polymorphism was found to be a risk factor in French Caucasian [309] population, whereas another study found association with the anti-centromere antibody and anti-topoisomerase I antibody positive subsets of the disease [310,311,312,313]. Other groups, however, found no evidence of association between SSc and R620W polymorphism in Spanish, Columbian and French patients [314,315,316], and other variants (R263Q and G788A) were not identified as risk factors either [313,317]. On the other hand, comparison of plasma samples obtained from 59 Italian SSc patients and 28 age- and sex-matched healthy volunteers revealed an elevated 2-AG level in the plasma of SSc patients [318]. Although these data are definitely not more than mere indirect pointers indicating the putative involvement of eCB dysregulation in SSc, additional evidence suggests that certain cannabinoids may have therapeutic value in this disease [319,320].

First of all, expression of CB_1_ and CB_2_ was already demonstrated in human dermal fibroblasts. Moreover, following a 24-h incubation, both UVA (20 J/cm^2^) and UVB (200 mJ/cm^2^) irradiation decreased the levels of AEA and 2-AG, but increased the expression of CB_1_, CB_2_, GPR55 and TRPV1 in human “CCD 1112Sk” foreskin fibroblasts [321]. Interestingly, both UVA and UVB irradiation appeared to alter cellular distribution of CB_1_, CB_2_ and TRPV1, increasing membrane, and decreasing cytosolic fractions of the receptors [204].

Up-regulation of CB_1_ and CB_2_ by pro-inflammatory challenges was further evidenced by LPS-treatment (10 μg/mL; 24 h). Importantly, in this system biological effects of the receptors were also tested by the co-administration of JWH-015, which was found to partially suppress the LPS-induced pro-inflammatory response in a CB_1_ and CB_2_ dependent manner [185], inviting the hypothesis that CB_1_/CB_2_ activators may exert beneficial anti-inflammatry effects in SSc. However, several additional studies have challenged this simplistic theory, arguing that the eCB-signaling may play a more complex regulatory role in vivo.

Indeed, expression of FAAH (more precisely: FAAH1) was found to be decreased in dermal cells (morphologically characterized to be fibroblasts) of SSc patients [322]. Furthermore, FAAH^−/−^ C57Bl/6 mice with strongly increased levels of eCBs were more sensitive to bleomycin-induced fibrosis than wild-type animals, as revealed by higher myofibroblast count and hydroxyproline content, as well as by more pronounced dermal thickening [322]. Consistently, pharmacological inhibition of FAAH-activity by JNJ 1661010 (4 mg/kg four times a day, i.p.) significantly exacerbated bleomycin-induced fibrosis. Of great importance, CB_1_ (AM281; 10 mg/kg four times a day, i.p.), but not CB_2_ (AM630; 2.5 mg/kg four times a day, i.p.), antagonism completely abrogated the pro-fibrotic effects of FAAH inhibition [322].

At this point, an important controversy has to be mentioned with respect to the expression of FAAH in human fibroblasts. In contrast to the above findings, a recent study (describing that a missense polymorphism [A458S] of FAAH2 may contribute to the development of psychiatric disorders including anxiety and mild learning disability) found that human dermal fibroblasts only express FAAH2, but not FAAH1 [64]. Since FAAH2 is not expressed in mice and rats, but shares the substrate spectrum of FAAH1 (however, it has less affinity towards AEA and N-acyl taurines), and conventional FAAH-inhibitors can inhibit its activity [49], targeted studies are invited to determine the expression patterns and putative roles of FAAH1 and FAAH2 in human fibroblasts under physiological as well as pathological conditions.

Irrespective of the expression pattern of FAAH1 and-2, CB_1_ appears to play a rather pro-fibrotic role in vivo, and could theoretically become a promising pharmacological target, especially, since CB_1_ (as well as CB_2_ [323] and TRPV4 [324]) were reported to be over-expressed in cultured lesional fibroblasts of patients suffering from diffuse cutaneous systemic sclerosis (dcSSc) compared with healthy controls [323]. However, since the authors did not provide appropriate densitometry analyses, the apparent alterations in the level of the loading control β-actin question the validity of this conclusion [323].

In line with the above observations, bleomycin-treatment induced less dermal thickening in TRPV4^−/−^ [324] as well as CB_1_^−/−^ mice as compared to wild-type animals. Moreover, activation of CB_1_ by the selective agonist ACEA (intraperitoneal injections twice a day at a concentration of 7.5 mg/kg for 4 weeks) further worsened bleomycin-induced dermal thickening. When assessing the mechanism of action, the authors found that, quite surprisingly, T cell and macrophage infiltration was significantly reduced in CB_1_^−/−^ mice following bleomycin challenge; whereas ACEA treatment could further increase it in wild-type animals. Last, but not least, the phenotype of CB_1_^−/−^ mice was mimicked by transplantation of CB_1_^−/−^ mouse bone marrow into CB_1_^+/+^ mice, demonstrating that CB_1_ exerted its pro-fibrotic effects indirectly by regulating infiltrating leukocytes. These data suggested that CB_1_ played a key role in positively regulating leukocyte infiltration in bleomycin-induced fibrosis in C57BL/6 mice [325]. This concept was further supported by additional evidence obtained in the non-inflammatory TSK-1 (“tight-skin”) mouse model of SSc model. TSK-1 mice carry a dominant mutation in the fibrillin 1 gene leading to accumulation of collagen fibers in the hypodermis, and thereby to progressive hypodermal thickening. In contrast to the aforementioned bleomycin-induced fibrosis, TSK-1 lacks inflammatory infiltrates, therefore abnormal fibroblast activation is not dependent on the release of inflammatory mediators from various immune cells [326]. Of great importance, lack of CB_1_ did not prevent fibrosis in the inflammation-independent TSK-1 mouse model, highlighting that CB_1_ signaling of the infiltrating immune cells is crucial in the development of bleomycin-induced fibrosis [325].

On the other hand, another study revealed that the role of CB_1_ is very likely to be even more complex. In fibroblasts isolated form SSc patients, adenosine A_2A_ receptors were found to be overexpressed, and the A_2A_ receptor antagonist ZM-241385 (1 μM; 24 h) could suppress pathologically elevated α-SMA expression of these cells [75]. Moreover the selective A_2A_ receptor agonist CGS-21680 (1 μM; 24 h) increased collagen production, and myofibroblast trans-differentiation (as monitored by α-SMA expression) both in healthy and in SSc fibroblasts, most likely via activating the ERK1/2 MAPK pathway [75]. Collectively, these data strongly argue that abnormally increased activity of A_2A_ may contribute to the pathogenesis of SSc [75]. Of great importance, A_2A_ receptor was found to heteromerize with CB_1_ (co-immunoprecipitation) in healthy as well as in SSC fibroblasts. Interestingly, although high (10 μM) concentration of the non-selective CB_1_ and CB_2_ agonist WIN55,212-2 suppressed collagen synthesis, its lower concentrations (when applied alone) had no effect on it. On the other hand, the combination of WIN55,212-2 and ZM-241385 (1 μM both) suppressed collagen production of SSc fibroblasts. Since, when applied alone at 1 μM, none of the compounds influenced collagen production, the authors concluded that by blocking A_2A_, ZM-241385 most likely indirectly antagonized its functional heteromer (i.e., CB_1_) as well, thus the remaining suppressive effect might have been coupled to the activation of CB_2_. Indeed, the CB_2_ antagonist/inverse agonist AM630 could prevent this effect at an unexpectedly low (1 nM) concentration, whereas its higher concentrations (5–1000 nM) had no effects, or could further enhance (20–80 μM) the actions of the WIN55,212-2+ZM-241385 combination [75]. Since AM630 was reported to be a “protean” ligand, i.e., under certain conditions (e.g., following 24-h pre-incubation of the cells with 10 μM SR144528, another CB_2_-selective inverse agonist) it may behave not only as an *antagonist/inverse agonist*, but also as a *low potency* (>25 μM) *agonist* at CB_2_ [327], the authors speculated that the latter phenomenon was the consequence of a putative paradoxical CB_2_-activating effect of AM630 [75]. Taken together, despite of the lack of certain key control experiments (e.g., determination of the percentage of co-localization/heteromerization of CB_1_ and A_2A_; assessment of the effects of CB_1_ and CB_2_ selective agonists; reversal of the effects of the A_2A_ agonist by a selective CB_1_ antagonist/inverse agonist), this study added an important new layer to the complexity of eCB-signaling [75], highlighting that, besides the effects on various immune cells, direct actions on fibroblasts may also be important.

Having dissected CB_1_, it is noteworthy that the role of CB_2_ was further investigated in a skin excisional wound model of BALB/c mice. The animals were treated with either the CB_2_ agonist GP1a, or with the antagonist AM630 (both in 3 mg/kg/day i.p.), where GP1a and AM630 induced opposing cellular effects. GP1a decreased collagen deposition, reduced the levels of TGF‑β1, TGF‑β receptor I and phosphorylated mothers against decapentaplegic homolog 3 (p‑Smad3), but elevated the expression of its inhibitor, Smad7, whereas AM630 increased collagen deposition and the expression levels of TGF‑β1, TGF‑β receptor I and p‑Smad3. Although the authors did not assess the effects of co-treatments, these results indicated that CB_2_ can modulate fibrogenesis and the TGF‑β/Smad profibrotic signaling pathway during skin wound repair in BALB/c mice [328].

Similarly, in a hypochlorite-induced BALB/c mice fibrosis model, WIN-55,212 (CB_1_ and CB_2_ agonist) and JWH-133 (a selective CB_2_ agonist) prevented the development of skin and lung fibrosis, and reduced fibroblast proliferation as well as the development of anti-DNA topoisomerase I autoantibodies. Experiments performed in CB_2_^−/−^ mice revealed that hypochlorite administration in these animals led to earlier and enhanced development of lung fibrosis and higher skin fibroblast proliferation rate. Moreover, CB_2_^−/−^ mice exhibited higher anti-DNA topoisomerase I autoantibody levels, and higher increase in splenic B cell count than wild-type animals [329]. Finally, CB_2_^−/−^ mice were more sensitive to bleomycin-induced dermal fibrosis than wild-type animals. Importantly, the phenotype of CB_2_^−/−^ mice was mimicked by transplantation of CB_2_^−/−^ bone marrow into wild-type animals, whereas CB_2_^−/−^ mice transplanted with bone marrow from CB_2_^+/+^ mice did not exhibit an increased sensitivity to bleomycin-induced fibrosis, indicating that CB_2_ expressed by the leukocytes is crucially important in this model of experimental fibrosis as well [330].

Along these lines, several exocannabinoids were also assessed in SSc. Ajulemic acid (AJA; a.k.a. CT-3, IP-751, JBT-101, anabasum or lenabasum) is a synthetic, cannabinoid-derived, orally bioavailable PPARγ and CB_2_ receptor activator, which has already been shown to exert remarkable anti-inflammatory and anti-fibrotic effects in various systems [319,320,331]. The effects of AJA included, but were not limited to prevention of bleomycin-induced dermal fibrosis, and a modest reduction in its progression when started 3 weeks after the onset of the symptoms. Moreover, AJA strongly reduced collagen production by SSc fibroblasts in vitro in a PPARγ-dependent manner [332]. Importantly, AJA showed anti-fibrotic efficiency in case of both “preventive” (i.e., administered from Day 0) and “therapeutic” (i.e., administered from post-bleomycin application Day 8) treatment in a DBA/2 mice model of lung fibrosis [333].

Encouraged by the above promising preclinical data, clinical investigation of AJA was also initiated. A multicenter, double-blind, randomized, placebo-controlled phase II trial assessing AJA efficiency in subjects with dcSSc was recently completed (ClinicalTrials.gov ID: NCT02465437). In this study, the AJA group showed higher Combined Response Index for Systemic Sclerosis (CRISS) score (i.e., greater improvement) as compared to the placebo group, suggesting that AJA may have potential as a novel drug in the management of SSc. Importantly, a phase III multicenter, double-blind, randomized, placebo-controlled study has already been announced (ClinicalTrials.gov ID: NCT03398837) to assess the efficacy and safety of AJA (lenabasum) for the treatment of dcSSc. Approximately 354 subjects are planned to be enrolled in this study at about 60 sites in North America, Europe, Australia, and Asia; the planned treatment duration is 1 year. Moreover, it is also noteworthy that efficiency and safety of AJA is currently assessed in some other diseases (namely dermatomyositis and cystic fibrosis) as well, and certain pre-clinical data suggest that it may exert beneficial effects in rheumatoid arthritis and multiple sclerosis too (summarized in [319]).

Besides AJA, certain CBD-derivatives also exhibited promising potential in SSc. Indeed, another PPARγ and CB_2_ co-activator (and CB_1_ antagonist), namely “VCE-004.3” (a semi-synthetic CBD quinol derivative) was also found to alleviate bleomycin-induced scleroderma as well as exerting potent anti-fibrotic effects via activating PPARγ and CB_2_ [242]. Similarly, another PPARγ- and CB_2_-activating CBD aminoquinone (VCE-004.8) could inhibit TGFβ-induced Col1A2 gene transcription and collagen synthesis, as well as TGFβ-induced myofibroblast differentiation, and it also impaired wound-healing. In bleomycin-induced fibrosis, VCE-004.8 reduced dermal thickness and collagen accumulation around blood vessels, it prevented degranulation of MCs, infiltration and activation of macrophages, as well as infiltration of T-cells. In addition, VCE-004.8 abrogated the bleomycin-induced up-regulation of several key genes associated with fibrosis (e.g., Col3A1, Col1A2, IL-1β and IL-13) [243]. Of great importance, EHP-101, an oral lipid formulation of VCE-004.8, was found to alleviate bleomycin-induced skin and lung fibrosis. Indeed, EHP-101 (25 mg/kg p.o.) prevented macrophage infiltration and dermal thickening, it suppressed vascular cell adhesion molecule 1 (VCAM1), tenascin C as well as α-SMA expression, and it normalized vascular CD31 positivity [334]. Moreover, RNAseq analysis of skin biopsies demonstrated that EHP-101 influenced inflammatory as well as epithelial-mesenchymal transition transcriptomic signatures. Indeed, bleomycin-induced alterations of several TGF-β-regulated genes (e.g., matrix metalloproteinase-3, cytochrome b-245 heavy chain, lymphocyte antigen 6E, VCAM1 and integrin alpha-5) were reversed by EHP-101 treatment. Moreover, EHP-101 could reduce expression of key SSc biomarker genes e.g., C-C motif chemokine 2 (CCL2) or the interleukin 13 receptor subunit alpha 1 (IL-13Rα1). Collectively, these data strongly argue that VCE-004.8 containing formulations deserve further attention as orally active agents to alleviate symptoms of SSc and maybe other fibrotic diseases as well [334].

With respect to the non-CB_1_/non-CB_2_ cannabinoid-activated pathways, it should also be noted that administration of WIN55,212-2 (1–10 μM) reduced expression of TGF-β and CTGF, as well as deposition of the extracellular matrix, and suppressed transdifferentiation of scleroderma fibroblasts into myofibroblasts and abrogated resistance to apoptosis. The anti-fibrogenic effect of WIN55,212-2 most likely involved inhibition of the ERK1/2 MAPK pathway, but, surprisingly, could not be prevented by selective CB_1_ and CB_2_ antagonists [323]. Anti-fibrotic effects of WIN55,212-2 were further dissected in another study. Here, co-treatment with WIN55,212-2 (1 mg/kg/day s.c.) prevented skin fibrosis in a DBA/2J mouse model of bleomycin-induced scleroderma. Administration of WIN55,212-2 prevented bleomycin-induced fibroblast activation (monitored by α-SMA positivity) and subcutaneous adipose tissue atrophy, suppressed subcutaneous infiltration of various immune cells, and reduced dermal fibrosis, as well as epidermal hypertrophy. Moreover, it decreased TGF-β, CTGF and PDGF-BB expression, and inhibited phosphorylation of SMAD2/3 [335]. Thus, further, targeted studies are necessary to unveil the exact mechanism of the potential anti-fibrotic effects of WIN55,212-2.

Last, but not least, it should also be noted that TRIB3, a potential cannabinoid target gene, was recently found to be greatly overexpressed in SSc fibroblasts, as well as in mice fibroblasts following bleomycin challenge [336]. Moreover, it was also demonstrated that breaking the TRIB3↔ TGF-β/Smad self-activating positive feedback loop by TRIB3 knock-down exerted potent anti-fibrotic effects [336]. Considering that, in human sebocytes CBD up-regulated TRIB3 in an A_2A_ receptor-dependent manner [120], and that A_2A_ receptors were found to be overexpressed in SSc fibroblasts [75], further studies are invited to dissect if dysregulation of the putative A_2A_↑→TRIB3↑ pro-fibrotic pathway plays a role in the pathogenesis of SSc. Thus, just like in PSO, up-regulation of TRIB3 appears to be undesirable. Intriguingly, however, down-regulation of another CBD target gene, namely NRIP1 (deletion of which in mouse embryonic fibroblasts suppressed fibroblast proliferation, enhanced autophagy, and delayed oxidative and replicative senescence [337]) promises to exert beneficial effects.

Taken together, these findings indicate that activation of CB_2_ and/or PPARγ as well as antagonism of CB_1_ and/or A_2A_ adenosine receptors may become potent tools in the management of SSc and maybe in other fibrotic diseases as well. Thus, systematic studies are invited to explore the putative therapeutic potential of cannabinoids characterized by such “molecular fingerprints”. Such cannabinoids may include pepcan-12 (a negative allosteric modulator of CB_1_, but a positive allosteric modulator of CB_2_ [338]), or THCV, which (albeit the available data about its pharmacology are somewhat controversial) was reported to be CB_1_ antagonist and CB_2_ agonist [57]). However, use of pCBs, which have the capability to activate the potentially pro-inflammatory TRPV3 [119,121] or the pro-fibrotic TRPV4 [324,339] ion channels could even be detrimental. On the other hand, since activation of TRPV1 expressed on the sensory nerve fibers was shown to be beneficial in SSc because of the release of certain sensory nerves-derived neuropeptides, e.g., calcitonin gene-related peptide [340], it seems to be almost unpredictable what the net effect of TRPV-activating pCBs would be in SSc. Systematic studies are therefore invited to explore putative therapeutic potential of these compounds in SSc and maybe in other fibrotic diseases too.

Putative inflammation-related translational potential of the cannabinoid signaling modulation is summarized in Table 5.

### 2.6. Wound Healing

Considering that cannabinoid signaling regulates fibroblast functions, proliferation and differentiation of epidermal keratinocytes, as well as cutaneous inflammation, it is not surprising that it influences the complex [341,342,343,344,345] process of cutaneous wound healing as well.

Murine data obtained after skin incision suggested that the expression pattern of CB_1_ [346] and CB_2_ [131] can be characterized by dynamic alterations during wound healing in various immune cells as well as in fibroblasts/myofibroblasts. Besides this, several additional lines of evidence support the concept that CB_1_ and especially CB_2_ can influence wound healing.

First, as mentioned above, in the presence of LPS, JWH-015 promoted wound closure in a scratch assay of human keratinocyte-fibroblast co-culture in a CB_1_ and CB_2_-dependent manner [185]. Moerover, VCE-004.8 (a PPARγ/CB_2_ dual agonist) was found to inhibit TGFβ-mediated myofibroblast differentiation, and to concentration-dependently (1–10 μM) impair human dermal fibroblast migration in a scratch assay [243]. Likewise, in a skin excisional model of BALB/c mice the CB_2_ agonist GP1a markedly attenuated fibrogenesis, whereas the CB_2_ blocker AM630 enhanced fibrotic events during skin wound healing via regulating the TGF‑β/Smad pro-fibrotic signaling pathway [328]. Intriguingly, however, by using the same CB_2_ agonist-antagonist pair, others have shown that CB_2_ agonism promoted migration of HaCaT keratinocytes in vitro, and enhanced re-epithelization in vivo in a BALB/c mice excisional wound model (3 mg/kg daily, i.p.), by inducing partial epithelial to mesenchymal transition [347]. Theoretically, such a dual effect (i.e., promotion of keratinocyte migration together with suppression of fibroblast activity) could be desirable to achieve scarless healing.

It should also be noted that abrogation of FAAH activity was found to accelerate skin wound healing in mice. Moreover, it stimulated migration of human keratinocytes, as well as differentiation of human fibroblasts to myofibroblasts. Intriguingly, however, these effects were not coupled to the elevated eCB-tone, but rather to an increase in the level of certain N-acyl taurines, and the subsequent (most likely indirect) activation of TRPV1 and epidermal growth factor receptor [348].

Topically applied platelet-rich plasma (PRP) [349] is widely used in regenerative medicine, since it improves tissue repair, and exerts potent analgesic effects [350]. In a recent study, administration of 5% (*v/v*) PRP pooled from ≥10 donors was shown to induce IL-8 and neutrophil gelatinase-associated lipocalin (NGAL) release from human NCTC 2544 keratinocytes via the activation of the RelA/p65 NF-κB pathway. Moreover, it has also been shown that PRP contained AEA, 2-AG, PEA and OEA, and that PRP-treatment induced AEA, 2-AG and OEA release from the keratinocytes. Of great importance, local administration of PRP before formalin injection into the hind paw of mice reduced the early response of the formalin-evoked nociceptive behavior by 42%, and completely abolished the late response. This anti-nociceptive effect was abrogated by local administration of CB_1_ (AM251), CB_2_ (AM630), and TRPV1 (I-RTX) blockers [350]. These data suggest that the clinically observed beneficial effects of PRP might be in part mediated through the ECS.

Interestingly, although potentially cannabinoid-responsive TRP channels are known to be involved in regulating several aspects of cutaneous (patho)physiology, including keratinocyte and fibroblast functions, barrier formation and regeneration, inflammation, etc. [3,45,94,351,352,353], only scant data are available with respect to cutaneous wound healing. Indeed, activation of TRPV3 with the combination of 1 mM camphor and 100 μM 2-APB induced NO production in cultured primary murine keratinocytes, which facilitated keratinocyte migration, and improved wound healing in mice [354]. On the other hand, TRPV2 antagonists (e.g., tranilast) may be efficient in preventing hypertrophic scar formation and contractures [355,356].

With respect to the efficiency of pCBs, only scarce evidence is available. Importantly, as mentioned above, three patients suffering from epidermolysis bullosa reported faster wound healing following self-administration of CBD [183]. Besides this, it should also be noted that a flax fiber-derived “CBD-like” compound as well as other bioactive substances in the flax fiber extract may promote wound healing, as they exerted anti-inflammatory activity, promoted migration of human keratinocytes and fibroblasts, and enhanced collagen production [357,358]. Thus, further studies are needed to assess putative efficiency of well-selected TRPV-modulating pCBs in cutaneous wound management. Putative wound healing-related translational potential of the cannabinoid signaling modulation is summarized in Table 6.

### 2.7. Itch

According to the definition of the German physician Samuel Hafenreffer, itch is an “unpleasant sensation that elicits the desire or reflex to scratch.” Pruritus, especially when it becomes chronic (>6 weeks), can severely impair quality of life. Although our understanding regarding its mechanism has grown a lot in the past years, there are still quite a few open questions [359]. Obviously, it would be far beyond the scope of the current paper to overview the pathogenesis of pruritus in details, especially, since comprehensive overviews have been published recently about itch in general [215,359,360], as well as about the role of various (mostly cannabinoid-responsive) TRP channels in its development [94,95,361,362,363]. Indeed, among others, all ionotropic cannabinoid receptors (i.e., TRPV1-4, TRPA1, and TRPM8) have been shown to play a role in the complex cutaneous intercellular communication network between epidermal keratinocytes, immune cells (e.g., MCs) as well as sensory nerves leading to itch sensation [94,95,361,362,363]. Thus, antagonizing or desensitizing such TRP channels by well-selected topically applied pCBs may hold out the promise of alleviating pruritus. Clinical trials are therefore invited to exploit putative therapeutic efficiency of topically applied, carefully selected pCBs in itch.

With respect to the effects of the “classical” ECS and to its related mediators, much less evidence is available. On one hand, “rs12720071”, “rs806368”, “rs1049353”, “rs806381”, “rs10485170”, “rs6454674”, and “rs2023239” polymorphisms of CB_1_ were not associated with uremic pruritus [364], but the synthetic THC analogue dronabinol (5 mg at bedtime) was reported to decrease pruritus for 4–6 h in 3 patients suffering from intractable cholestatic itch [365].

The latter preliminary data suggested that the ECS and CB_1_ may have anti-pruritic activity. However, especially in case of CB_1_ modulation, one has to carefully differentiate between behavioral effects exerted via activating/antagonizing central nervous system CB_1_, and peripheral, partially non-neuronal actions. Indeed, i.p. administration of the CB_1_ antagonist/inverse agonist rimonabant (SR141716A) induced head scratching behavior in mice, which could be prevented by the 5-HT_2A_/5-HT_2C_ antagonist ketanserin [366]. However, this effect was likely to be rather a central than a peripheral action of rimonabant, since LH-21 (another CB_1_ antagonist with relatively poor brain-penetration) did not induce head scratching behavior [367]. In line with these observations, intraperitoneally administered WIN55,212-2 (1–10 mg/kg) dose-dependently suppressed scratching in BALB/c mice, which were intradermally injected with 5 μg/50 μL serotonin. Importantly, the intrathecally applied CB_1_ antagonist/inverse agonist AM251 (1 μg), but not the CB_2_ antagonist/inverse agonist AM630 (4 μg), could partially prevent anti-pruritic effects [368], indicating that activation of spinal CB_1_ may possess anti-pruritic activity.

Besides the above data, certain reports argue that not only brain and spinal, but also peripheral CB_1_ may be a potent contributor in itch. Indeed, as mentioned above, RNAseq of the skin of AD and PSO patients suffering from severe itch revealed that CB_1_ and CB_2_ were significantly down-regulated in both diseases indicating that loss of homeostatic cutaneous CB_1_/CB_2_ signaling may disease-independently contribute to the development of chronic itch [276]. Moreover, 24-h pre-treatment (in 8 mm Finn Chambers following tape-stripping) with HU-210 (a highly potent agonist of CB_1_ and CB_2_, capable of activating GPR55, and modulating glycine receptors as well [369]; 50 μL of 50 mM solution) suppressed histamine-induced scratch in human volunteers [370].

Intriguingly, the available evidence is somewhat controversial with respect to the role of CB_2_. The CB_2_ specific inverse agonist JTE-907 (1 and 10 mg/kg/day p.o.) was found to reduce spontaneous itch-associated responses in NC mice [295], whereas the orally administered novel CB_2_ agonist S-777469 also suppressed itch (induced by histamine or substance P in mice or by serotonin in rats)-associated scratching behavior in rodents. Indeed, scratching was reduced in a CB_2_-dependent manner, since oral pretreatment with the CB_2_ antagonist SR144528 could prevent the effect [371]. Considering that S-777469 inhibited histamine-induced nerve firing, the authors concluded that it elicited anti-pruritic effects via inhibiting itch signal transduction by activating CB_2_ expressed on the peripheral sensory nerve fibers [371].

Importantly, elevation of the eCB-tone by PF-3845 (FAAH-inhibitor; 5, 10, and 20 mg/kg, i.p. or 1, 5, and 10 µg, i.t.), JZL184 (MAGL-inhibitor; 4, 20, and 40 mg/kg, i.p. or 1, 5, and 10 µg, i.t.), as well as JZL195 (a dual FAAH/MAGL-inhibitor; 2, 5, and 20 mg/kg, i.p. or 1, 5, and 10 µg, i.t.) exerted potent spinal anti-pruritic effects in a serotonin-induced pruritus model of BALB/c mice [372]. Partially in line with these observations, another FAAH-inhibitor (URB597; 10 mg/kg, i.p.), as well as the aforementioned JZL184 (16 mg/kg, i.p.), but, intriguingly, not the EMT-inhibitor AM404 (10 mg/kg, i.p.), were found to attenuate serotonin-induced scratches in the same model system. Interestingly, anti-pruritic effects of URB597 (but not of JZL184) could be reversed by the CB_2_ antagonist SR144528 (1 mg/kg, i.p.), whereas the CB_1_ selective antagonist/inverse agonist AM251 (1 mg/kg, i.p.) had no effects [373]. Since AM404 may concentration-dependently inhibit other targets (e.g., FAAH) as well, future studies using selective EMT-inhibitors (e.g., WOBE437 [42,374]) are invited to confirm or refute existence of such functional differences between the effects of EMT- and FAAH/MAGL-inhibitors.

The anti-pruritic efficiency of FAAH-blockade was further shown in an allergenic model of pruritus [375]. Indeed, subcutaneous administration of compound 48/80 (30 μg; a well-known inducer of MC degranulation) evoked an intense, concentration-dependent scratching response. Pre-treatment with THC (1–3 mg/kg; i.p.) reduced the scratching response in a CB_1_-dependent manner, although this effect was accompanied with hypomotility, i.e., it might have been an artifact. Of great importance, compound 48/80-induced scratching was reduced without influencing motility in global FAAH^−/−^ mice, as well as by administration of FAAH-inhibitors (URB597 and OL-135) in wild-type, again, in a CB_1_-dependent manner. Finally, experiments conducted on “FAAH-NS” mice (conditional knockin mice with FAAH expression linked to the promoter for neuron-specific enolase, resulting in mice that express FAAH exclusively in neuronal tissues) revealed that neuronal FAAH expression is enough to restore scratching behavior [375].

Finally, it should also be noted that eCBs may exert opposing actions on scratching behavior in trigeminally- and spinally-innervated skin [376]. Indeed, locally injected URB597 and JZL184 suppressed serotonin-induced scratching in the rostral back in a CB_1_- and CB_2_-dependent manner in Sprague Dawley rats. In the cheek, however, URB597, JZL184 as well as AM630 enhanced scratching [376].

Having discussed the available evidence related to CB_1_ and CB_2_, the role of the ECS-related substance PEA should also be mentioned. Importantly, in a DNFB-induced contact allergic dermatitis model of C57BL/6J mice, PEA (5 mg/kg; i.p.) was found to reduce ear scratching in a CB_2_- and PPARα-dependent manner, since both AM630 and GW6471 (both at 1 mg/kg; i.p.) could prevent the effect [202]. Similarly, the NAAA-inhibitor ARN077 dose-dependently suppressed edema formation and scratching in DNFB-induced dermatitis likely by elevating local PEA levels, and subsequently activating PPARα. Likewise, DNFB induced significantly less scratching in NAAA^−/−^ mice compared to the wild-type animals [297], and as mentioned above, PEA-um was found to reduce itch in dogs with moderate AD and moderate pruritus [299].

Importantly, with respect to the efficiency of PEA, human clinical data are also available. Indeed, the PEA containing Physiogel^®^ A.I. Cream was found to alleviate itch in 14 out of 22 patients suffering from prurigo, lichen simplex and other pruritic diseases [377]. Importantly, the same formulation was found to be effective in alleviating erythema, excoriation, scaling, lichenification, dryness, as well as pruritus in AD patients (ATOPA study) [300]. However, another vehicle controlled, randomized clinical trial involving a total of 100 subjects suffering from pruritic dry skin (ClinicalTrials.gov ID: NCT00663364) found that a PEA containing lotion was not significantly superior in alleviating itch as compared to its emollient vehicle [378].

Putative pruritus-related translational potential of modulation of cannabinoid signaling is summarized in Table 7.

### 2.8. Skin Tumors

#### 2.8.1. General Considerations

It is well-known that medical marijuana has become increasingly popular as palliative treatment in case of various malignant tumors [379,380]. However, its superiority compared to other treatment modalities is not unambiguously confirmed yet; therefore, well-organized, placebo-controlled double-blind multicenter clinical trials with large sample sizes are necessary in order to find the most efficient way of using cannabis-based therapies [379,380,381]. On the other hand, a large body of evidence demonstrates that pharmacological modulation of the cannabinoid signaling may have direct anti-tumor effects beyond mere palliation. Indeed, although there are some controversial data (e.g., CB_1_ was found to promote growth of human A375 and 501 Mel melanoma cell lines [382]; CB_1_^−/−^/CB_2_^−/−^ double KO mice were protected against DMBA and UVB-treatment induced papilloma formation [87]), majority of the studies agree that cannabinoids deserve further attention as putative future anti-cancer drugs in general [383,384], and in the case of skin tumors as well [100].

Indeed, there are quite a few papers demonstrating that appropriate modulation of the complex cannabinoid signaling may exert anti-tumor activity in case of melanoma and non-melanoma skin cancers. However, when reviewing these data, two important general considerations must always be kept in mind. First, “the dose makes the poison” (Paracelsus), i.e., sufficiently high concentrations and/or long treatment durations will undoubtedly lead to anti-proliferative or pro-apoptotic effects in cell cultures irrespective of the test substance. Thus, data about cannabinoid-mediated in vitro anti-tumor effects at extremely high concentrations without in vivo confirmation should be interpreted very carefully. Besides this, another key issue in case of cannabinoid administration may be the suppression of the anti-tumor immune response [31,385]. Thus, in spite of any promising in vitro (or even in vivo data obtained in immune-deficient animal models) paradoxically, cannabinoid treatment might indirectly promote tumor growth in certain cases (obviously, most likely in those cases, when the tumor cells do not express cannabinoid receptors) [31].

#### 2.8.2. Melanoma

Although by using novel approaches (e.g., PD-1—PD-1L blockers) 5-year overall survival rates for metastatic melanoma have increased substantially from less than 10% to up to 40−50% [386], there is still an unmet need to further improve our therapeutic arsenal. Therapeutic exploitation of lipid (including eCB) signaling in melanoma is an intriguing, novel direction of the field [387]. This is especially true since certain data suggest that dysregulation of the homeostatic eCB-signaling may develop in melanoma.

A comparison of 20 melanoma and 20 non-melanoma patients revealed that expression of CB_2_ was up-regulated in melanomas as compared to nevi or normal melanocytes [388]. Moreover, as mentioned above, a recent study questioned the expression of MAGL in normal epidermal melanocytes, but demonstrated that it was present in melanoma cell lines. MAGL expression was found to positively correlate with tumor thickness, as well as with vascular invasion of the primary lesion and tumor progression, suggesting that strongly MAGL-positive tumors were more aggressive [157]. Although another group could identify MAGL expression in healthy melanocytes as well [155], these findings invite new studies exploring if MAGL-inhibitors (and e.g., the subsequently enhanced/restored CB_2_ signaling) may be effective in the management of cutaneous melanoma.

Interestingly, not only CB_2_ and MAGL, but also eCB-levels may be altered in melanoma. Indeed, in the plasma of 304 patients decreased AEA, whereas elevated OEA and 2-AG levels were found compared to healthy individuals. This may be somewhat surprising in light of the above MAGL data, but importantly, similar observations were made in a B16 cells-induced melanoma model of C57BL/6J mice, and the alterations correlated with the number of metastases [389].

When assessing the effects of the eCBs, AEA was found to by cytotoxic (IC_50_: 5.87 ± 0.7 μM) in human A375 melanoma cells in a CB_1_-, COX_2_-, and a caspase-dependent manner, but neither CB_2_, nor TRPV1 antagonists influenced the effect. Simultaneous FAAH-inhibition by URB597 (1 μM), however, enhanced cytotoxicity of AEA [390]. Moreover, the GPR55 agonist O-1602 also decreased viability (IC_50_ of 17.57 ± 2.6 μM). Of great importance, methyl-β-cyclodextrin, a membrane cholesterol depletor, could reverse the effects of AEA as well as of O-1602, suggesting that membrane lipid rafts and local lipid microenvironment of CB_1_ and GPR55 may play an important role in regulating activity of these receptors [390]. Similarly, PEA (1–20 μM; 72 h) was found to decrease viability of B16 mouse melanoma cells in a concentration-dependent manner, which was further enhanced by simultaneous FAAH, but intriguingly not by NAAA, inhibition [391]. A combination of PEA+URB597 (both at 10 mg/kg/day; i.p.) was also efficient in reducing tumor mass in vivo following subcutaneous injection of B16 cells to C57BL/6 mice [391]. Last, but not least, OEA (2–100 μM) was found to inhibit migration of B16 melanoma cells in scratch assay, but further promoted it at 10–500 nM [389], highlighting that in certain cases eCBs might exert concentration-dependently opposing effects.

Irrespective of this, the above data indicate that CB_1_ and GPR55 might be promising targets in the clinical management of melanoma. In line with these observations, the human A375 as well as the mouse B16 melanoma cell lines, together with human melanomas were found to express CB_1_ and CB_2_, activation of which by 100 nM WIN55,212-2 or 1 μM THC decreased viability of the cells (48–72 h) [392]. The effects could be prevented by the co-administration of SR141716 (rimonabant; CB_1_ antagonist/inverse agonist; 500 nM), SR144528 (CB_2_ antagonist; 500 nM) and AM630 (CB_2_ antagonist/inverse agonist; 1 μM) [392]. Importantly, WIN55,212-2 and THC did not influence viability of the non-tumorigenic human Hermes 2b and mouse melan-c melanocyte cells lines [392]. Following tumor formation by injecting B16 cells into C57BL/6 mice, peritumoral injections of WIN55,212-2 as well as of the CB_2_-selective agonist JWH-133 (both at 50 μg/day) suppressed growth, proliferation, angiogenesis and metastasis formation, but increased apoptosis of melanomas in vivo. The above anti-melanoma activity was independent of the immune status of the animals, and could be achieved via the inhibition of Akt signaling and hypophosphorylation of the retinoblastoma tumor suppressor protein [392]. However, treatment of the CB_2_ and (interestingly, mostly intracellular) CB_1_ expressing COLO38 (a melanoma-associated proteoglycan [MPG] antigen positive human melanoma cell line) and OCM-1 (non-metastatic human ocular choroidal melanoma cells) melanoma cells with WIN55,212-2 (2–5 μM) reduced viability in a CB_1_/CB_2_-independent manner, and induced phosphorylation of the ERK1/2 MAPK cascade (5 μM; 24 h). Importantly, the lipid raft disruptor methyl-β-cyclodextrin (1 mM) prevented both effects [393].

Partially in line with these data, CB_1_ mRNA was found to be expressed in 4 human melanoma cell lines (non-metastatic: WM35 or metastatic: HT168, A2068, HT168-M1), and confocal microscopy revealed that CB_1_ was expressed both at the cell membrane as well as in the cytosol of HT168-M1 cells [394]. AEA, 2-methyl-2-F-anandamide (met-F-AEA; a metabolically stable synthetic AEA analogue), ACEA (CB_1_ agonist) and AM251 (CB_1_ antagonist/inverse agonist) suppressed proliferation at the low micromolar range. Moreover, ACEA (0.24 mg/kg; i.p.) inhibited liver colonization of human HT168-M1 melanoma cells in SCID mice [394]. Considering that, besides antagonizing CB_1_, higher concentrations of AM251 can also activate GPR55 (and behave as a GPR18 partial agonist) [395], its anti-proliferative effects were likely to be coupled to the activation of GPR55, which has already been shown to mediate cytotoxic effects (see above [390]). Intriguingly, however, in a more recent study, CB_1_-silenced human A375 and 501 Mel melanoma cell lines exhibited reduced viability, colony-forming ability and cell migration, due to an arrest at G1/S phase, and suppressed expression of p-Akt and p-ERK1/2, which suggests that both (over)activation and complete loss of CB_1_ signaling may impair viability. Thus, the role of CB_1_ (with a special emphasis on its spatially distinct sub-populations) needs to be further explored in human cutaneous melanoma [382].

In another study, 1–10 μM AM251 (48–72 h) induced apoptosis and G2/M cell cycle arrest in A375 human melanoma cells in a GPR55-, TRPA1-, and COX_2_-independent manner, whereas the combination of AM251 with COX_2_-inhibitor celecoxib produced a synergistic antitumor activity [396]. Since, as mentioned above, besides CB_1_ and GPR55, AM251 can also target GPR18 [395], one might speculate that modulating activity of this receptor may be responsible for the beneficial effects, especially, since GPR18 (as well as GPR119) was found to be overexpressed at the mRNA level in melanomas as compared to nevi [397]. Importantly, siRNA-mediated silencing of GPR18 induced apoptosis in the human lymph node metastasis-derived Cmel 0709 melanoma cell line [397]. Further studies are therefore invited to explore if, as a partial agonist, AM251 can GPR18-dependently exert anti-melanoma activity.

Besides CB_1_ and GPR55, the role of CB_2_ was also investigated. Expression of CB_1_, CB_2_, GPR18, GPR55 and GPR119 were identified in A2058 human amelanotic melanoma cell line. Activation of CB_2_ by JWH-133 (10 μM) was found to reduce the transmigratory capability of A2058 cells through primary rat brain endothelial cells mimicking the blood-brain barrier [398], which means that CB_2_ agonists may be efficient in preventing brain metastasis formation.

As suggested by the above data, certain cannabinoid-responsive receptors may exert potent anti-tumor activity. Several TRP channels have also emerged as potential anti-tumor target molecules [139,399,400,401,402,403,404]. Unfortunately, skin-wise only scant evidence is available about the putative role of cannabinoid-responsive TRP channels in tumorigenesis [139,399,400,401,402,403,404,405]; further studies are therefore invited to unveil the putative therapeutic potential of pharmacological modulation of these molecules in melanoma and non-melanoma skin tumors.

With respect to the effects of pCBs, it should be noted that 24-h treatment with the CB_2_ activator β-caryophyllene decreased viability of C32 human amelanotic melanoma cells (IC_50_: 20.1 ± 0.4 μg/mL) [406]. Moreover, orally administered β-caryophyllene was found to inhibit solid tumor growth and lymph node metastasis of B16-F10 melanoma cells in high-fat diet-induced obese C57BL/6N mice. Unfortunately, however, the putative involvement of CB_2_ was not tested [407].

As mentioned above, 1 μM THC was found to decrease viability of A375 and B16 melanoma cell lines in a CB_1_- and CB_2_-dependent manner [392]. In contrast to this, another study found that up to 10 μM, THC had no effect on the growth and viability of the CB_1_/CB_2_ positive HCmel12 (established from a primary, 7,12-dimethylbenz(a)anthracene-induced melanoma of HGF-CDK4^R24C^ mouse [408]) and B16 melanoma cells in vitro as determined by trypan blue exclusion assay [392]. Importantly, THC (5 mg/kg/day; s.c.) did not influence tumor formation of transplanted B16 cells either. However, it significantly suppressed tumor growth of transplanted HCmel12 melanomas in wild-type animals, whereas it was ineffective in CB_1_^−/−^/CB_2_^−/−^ double KO mice. THC did not affect vascularization of the tumor, but a reduction in the number of infiltrating CD45+/CD11b+/Gr1- (dominantly macrophages) and CD45+/CD11b+/Gr1+ (dominantly neutrophils) immune cells was observed in wild-type animals, suggesting that certain, yet un-characterized, immunological effects of THC might contribute to its anti-melanoma activity [409].

Importantly, a few additional studies also argue that THC might exert anti-melanoma effects. Indeed, treatment with low micromolar THC concentrations decreased viability of human A375, SK-MEL-28, and CHL-1 melanoma cells, most likely via activating autophagy and subsequent apoptosis [410]. Intriguingly, administration of a “Sativex-like” preparation, which contained equal amounts of THC and CBD appeared to be even more efficient [410]. Of great importance, THC (15 mg/kg/day; p.o.) as well as a “Sativex-like” preparation (7.5 mg/kg/day THC-botanical drug substance [BDS] and equal amount of CBD-BDS, p.o.) were able to substantially inhibit melanoma viability, proliferation, and tumor growth in mice bearing BRAF wild-type melanoma xenografts (CHL-1 cells) [410]. The effects were comparable that of the standard alkylating agent temozolomide, and were paralleled by an increase in autophagy and apoptosis [410]. These findings suggested that THC activated a non-canonical autophagy-mediated apoptosis pathway, most likely via enhancing TRIB3 activity [410].

Intriguingly, although in light if the above data, activating TRIB3 promises to be a potent tool in suppressing melanoma progression, other data seem to contradict these findings. Namely, the anti-diabetic drug metformin (150 mg/kg/day; p.o.) was found to suppress melanoma (evoked by subcutaneous injection of B16-F10 cells) progression in non-diabetic C57BL/6 mice as well as in diabetic KK-Ay mice by inhibiting the lysine acetyltransferase 5 (KAT5)/TRIB3/SMAD3 positive feedback loop. Moreover, suppression of TRIB3 was found to restore autophagy flux; thus, these data suggested that down-regulating expression and/or inhibiting activity of TRIB3 may be a potent anti-melanoma strategy [411,412]. Further studies are therefore invited to explore the putative therapeutic potential of modulating TRIB3 expression/activity in melanoma.

#### 2.8.3. Non-Melanoma Skin Cancers

Several lines of evidence suggest that cannabinoid signaling may play a role in non-melanoma skin cancers as well. Indeed, CB_1_ and CB_2_ were shown to be expressed not only in human and mouse keratinocytes, but also in various tumors, namely chemically-induced mouse papilloma, as well as mouse and human squamous cell carcinoma (SCC) and human basal cell carcinoma (BCC) [133]. Moreover, according to another study, CB_2_ was found to be overexpressed in SCC both at the mRNA and at the protein levels [413]. These data, together with the fact that CB_1_ can exert anti-proliferative actions in human keratinocytes [174], suggested that CB_1_ and CB_2_ may exhibit certain anti-tumor actions in non-melanoma skin cancers as well.

WIN55,212-2 (25 nM; 3–4 days) was found to reduce viability (MTT-assay) and induce apoptosis (TUNEL assay) of HaCaT as well as of PDV.C57 cells (a tumorigenic mouse epidermal cell line), and the effect could be prevented by the co-administration of SR141716 (rimonabant; CB_1_ antagonist/inverse agonist; 0.2 μM) and SR144528 (CB_2_ antagonist/inverse agonist; 0.2 μM) [133]. Moreover, peritumoral administration of WIN55,212-2 as well as of the selective CB_2_ agonist JWH-133 could suppress tumor growth and angiogenesis in vivo in NMRI nude mice following subcutaneous flank inoculation of PDV.C57 epidermal tumor cells, most likely via suppressing epidermal growth factor receptor expression and activation (autophosphorylation) [133]. In line with these observations, in ICR mice, the CB_2_ activators JWH-018, JWH-122 and JWH-210 exhibited potent anti-inflammatory activity, and inhibited tumor promotion by TPA in a two-stage mouse skin carcinogenesis model [414]. Intriguingly, there was no difference in the development of chemically induced skin tumors (subcutaneous application of 3-methylcholanthrene) between wild-type and CB_1_^−/−^/CB_2_^−/−^ double KO mice [409], although the latter group exhibited significantly less skin papilloma formation following DMBA and UVB-treatment [87], suggesting that the role of CB_1_ and CB_2_ signaling in regulating tumor formation may be context-dependent.

Having discussed CB_1_ and CB_2_, it should also be noted that GPR55^−/−^ mice were more resistant to DMBA/TPA-induced papilloma and carcinoma formation than their wild-type littermates. In addition, GPR55 enhanced skin cancer cell anchorage-independent growth, invasiveness and tumorigenicity in vivo, suggesting that it may promote not only tumor development, but also tumor aggressiveness [415]. Importantly, in line with these observations, GPR55 mRNA was found to be up-regulated in human SCC, as well as in larynx and oral squamous cell carcinomas compared to the respective healthy tissues [415].

Finally, we have to mention that eCBs may be able to exert receptor-independent anti-tumor actions as well. In the murine squamous carcinoma cell line JWF2, AEA (20 μM) induced oxidative stress by reducing the intracellular level of glutathione [416]. Importantly, unlike antagonists of CB_1_, CB_2_ and TRPV1, antioxidants e.g., N-acetylcysteine and Trolox (6-hydroxy-2,5,7,8-tetramethyl- chroman-2-carboxylic acid; a vitamin E analog) could suppress the anti-proliferative effect. Moreover, Trolox could also prevent AEA-induced CHOP10 expression and caspase 3 activity, indicating that oxidative stress was required for AEA-induced ER stress-apoptosis [416]. Further scrutiny of the mechanism of action revealed that such ER-stress only occurred in the presence of COX_2_, most likely because this enzyme metabolized AEA to cytotoxic J-series prostaglandin-ethanolamides (prostamides) [417,418]. The putative anti-tumor therapeutic potential of cannabinoid signaling is summarized in Table 8.

## 3. Challenges, Open Questions, Promising Future Directions

### 3.1. Potential Side Effects

When talking about cannabinoid-based drug development, the most obvious challenge is that activation of CB_1_ can lead to cardiologic and psychotropic side effects, tolerance, dependence or even juvenile memory impairment [41]. Although the latter seems to be age-dependent (in fact, THC was shown to CB_1_-dependently *improve* memory function in aged mice [419], whereas lack of homeostatic CB_1_ signaling in aged CB_1_^−/−^ mice led to a premature decline in cognitive abilities [420]), it is important to emphasize that activation of mitochondrial, but not surface membrane, CB_1_ is responsible for THC-induced memory impairment [81,82]. Thus, extracellularly-restricted CB_1_ activators are likely to be devoid of such side effects.

Intriguingly, not only activation, but also antagonism/inverse agonism of brain CB_1_ can lead to severe psychiatric side effects (including suicide). This is the reason why the brain-penetrating CB_1_ inverse agonist rimonabant (“SR141716”; previously marketed as “Acomplia” and “Zimulti”), although a highly potent anorexigenic agent, had to be withdrawn from the market [421,422]. Thus, designing novel, peripherally acting CB_1_ antagonists/inverse agonists [423,424,425], as well as appropriate topical formulations delivering phyto- or other cannabinoids directly to the desired skin compartments (but, ideally, not to systemic circulation and especially not to the central nervous system) will be a key goal of future dermatological drug development [189,426].

Another central challenge of future drug development is the aforementioned complexity of the ECS (Figure 1 and Figure 2), including polypharmacology, biased agonism, heteromerization, context-dependence, etc. Rigorous pre-clinical testing and thorough exploration, investigation and evaluation of all compounds exhibiting therapeutic potential is clearly indispensable, as it is sadly exemplified by the tragic phase I clinical trial of “BIA 10-2474”, which led to the death of one volunteer and produced mild-to-severe neurological symptoms in four others [427,428]. “BIA 10-2474” was supposed to be a specific, novel FAAH-inhibitor; however, later it has been proven to be a highly unspecific lipase inhibitor, and its side effects most probably developed due to complex metabolic dysregulation in the central nervous system caused by unanticipated off-target effects [427,428].

### 3.2. Unidentified Players: Intercellular Transport, Cellular (Re-)uptake, Intracellular Trafficking

As discussed above, elevation (or more precisely: restoration) of the local eCB-tone promises to be a potent tool in a wide-variety of inflammatory skin diseases. Theoretically, this could be achieved by e.g., FAAH-inhibitors (leading to a primarily intracellular accumulation of the eCBs) as well as by blocking the putative EMT (most likely resulting in a primarily extracellular elevation in the eCB-levels). Although the two ways are similar, they may not be identical (see e.g., [373]). On one hand, EMT-inhibitors may be more selective in elevating eCB-tone, since FAAH-inhibitors could also increase the levels of other molecules, e.g., N-acyltaurines [49,348]. On the other hand, because of the site of the primary eCB elevation, the differing available target spectrum (surface membrane CB_1_ vs. mitochondrial or lysosomal CB_1_, PPARs, etc.) may lead to significant functional differences. Obviously, it would also be crucially important to understand further details of the regulation of inter- and intracellular trafficking of eCBs, since selective modulation of these pathways could also help in narrowing the target spectrum of the eCBs.

### 3.3. Identification of “Disease—Cannabinoid” Pairs

As mentioned above, perhaps the most important challenge is the remarkable complexity of the cannabinoid signaling due to, among others, the pharmacological promiscuity of the cannabinoids. However, one could take advantage of this issue by predicting and identifying “disease—cannabinoid pairs” (or “therapeutic handshakes” [429]). Indeed, without being exhaustive, THCV and/or pepcan-12 (both suppressing CB_1_ and promoting CB_2_ activity) could nicely match the therapeutic needs in SSc. Similarly, by exerting anti-inflammatory actions, slightly promoting SLG, and desensitizing the pro-inflammatory TRPV3, CBGV appears to be promising in AD. Identification of such pairs promises to greatly improve the efficacy of selecting candidate compounds for clinical testing.

## 4. Concluding Remarks

**Figure 3:**
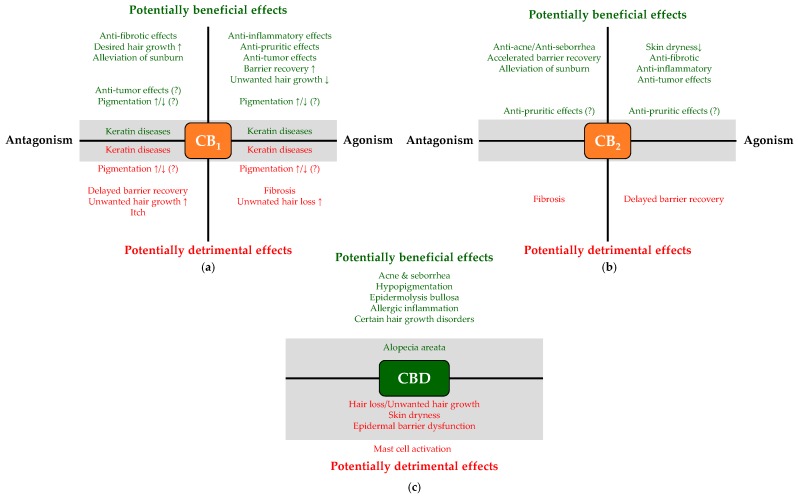
Schematic overview of potentially beneficial and detrimental consequences of pharmacological modulation of CB_1_ (**a**) and CB_2_ (**b**), as well as of CBD administration (**c**). Note that certain effects (e.g., promoting hair growth) can context-dependently be considered to be a beneficial (e.g., in hirsutism) or a detrimental (e.g., in alopecia) outcome. Question marks indicate controversial data, whereas gray background highlight unproven effects, which are only hypothesized based on indirect evidence; thus, systematic studies are invited to unveil if they indeed develop.

Although the most prevalent dermatological disorders are usually not directly life-threatening ones, their symptoms can dramatically impair quality of life of millions of patients world-wide. As discussed above, research efforts of the past two decades have undoubtedly proven that cannabinoid signaling profoundly influences several aspects of the cutaneous biology, and its dysregulation is likely to contribute to the pathogenesis of several skin diseases. Although, as briefly discussed above, a number of open questions await to be answered, appropriate pharmacological modulation of the cutaneous cannabinoid signaling promises to be a powerful tool in treating such diseases (Figure 3). Systematic basic research efforts as well as clinical trials are therefore invited to exploit the untapped potential of the cannabinoid system in managing skin diseases, in order to pave new “high”-ways towards developing novel therapeutic tools.

## Figures and Tables

**Table 1:** Overview of the putative sebaceous gland-relevant therapeutic potential of cutaneous cannabinoid signaling.

Disease	Intervention	Level of Evidence	References
Dry skin	EMT-inhibition (elevation of the eCB-tone)	In vitro (cell culture) data	[112,114]
	CBG, CBGV	In vitro (cell culture) data	[126]
Acne & Seborrhea	CBD (via activating TRPV4 and A_2A_ receptors)	In vitro (cell culture) and ex vivo (organ culture) data	[120]
	BTX 1503 (synthetic CBD containing cream)	Successful phase Ib and ongoing phase II clinical trials	[125] ClinicalTrials.gov ID: NCT03573518
	THCV, CBC, CBDV	In vitro (cell culture) data	[126]
	3% *Cannabis* seeds extract cream	single-blind, split-face study	[124]
	Reduction of the eCB-tone	In vitro (cell culture) data	[112,114]
	GPR119-antagonism ^1^	**Hypothesis** based on preliminary in vitro (cell culture) data	[114,116]

^1^ Note that effects of GPR119 antagonism have not been tested yet; however, in light of the available scarce data, interfering with GPR119 signaling might deserve systematic experimental exploration.

**Table 2:** Overview of the putative hair-relevant therapeutic potential of cutaneous cannabinoid signaling.

Disease	Intervention	Level of Evidence	References
Unwanted hair growth (hirsutism, hypertrichosis)	Certain CB_1_ agonists	Ex vivo (organ culture) data	[130]
	TRPV1, TRPV3 and TRPV4 activators	Ex vivo (organ culture) data	[135,136,137,138]
Unwanted hair loss (different non-immune alopecia forms)	Certain CB_1_ antagonists/inverse agonists	Ex vivo (organ culture) and in vivo (mouse) data	[130,134]
	TRPV1, TRPV3 and TRPV4 antagonists	Ex vivo (organ culture) data	[135,136,137,138]
Alopecia areata	Elevation of the eCB-tone; certain CB_1_ agonists, low doses of CBD ^1^	**Hypothesis** based on the available data	[31,130,140,146,147,148,149,150,151,152,154]

^1^ Note that well-controlled studies proving the efficiency of the indicated interventions are missing; however, in light of the available data, cannabinoid signaling might exert beneficial effects in alopecia areata, therefore it deserves systematic experimental exploration.

**Table 3:** Overview of the putative pigmentation-relevant therapeutic potential of cutaneous cannabinoid signaling.

Disease	Intervention	Level of Evidence	References
Hypopigmentation	Elevation of the eCB-tone/activation of CB_1_ (?)	In vitro (monoculture of primary human epidermal melanocytes)	[155]
	Administration of CBD (via activating CB_1_)	In vitro (monoculture of primary human epidermal melanocytes)	[60]
Hyperpigmentation	Elevation of the eCB-tone/activation of CB_1_ (?)	In vitro (co-culture of SK-mel-1 and HaCaT keratinocytes)	[160]
	β-caryophyllene	In vitro (mono-culture of B16 melanoma cells)	[158]
Vitiligo	Elevation of the eCB-tone ^1^	**Hypothesis** based on literature data	[164,165,166,167]

^1^ Note that well-controlled studies proving the efficiency of cannabinoids are missing; however, in light of the available data, elevation of the eCB-tone might exert beneficial effects in vitiligo, therefore it deserves systematic experimental exploration.

**Table 4:** Overview of the putative keratinocyte-relevant therapeutic potential of cutaneous cannabinoid signaling.

Disease	Intervention	Level of Evidence	References
Epidermolysis bullosa	Topical CBD	Case report of 3 patients	[183]
	Sublingual THC and CBD containing CBM oil	Case report of 3 patients	[184]
Pachyonychia congenita	ACEA (and maybe other CB_1_ agonists)	Ex vivo (hSOC)	[175]
Epidermolytic ichthyosis	ACEA (and maybe other CB_1_ agonists)	Pilot ex vivo (hSOC)	[176]
Barrier disruption	CB_1_ activation and/or CB_2_ blockade	In vivo (CB_1_^−/−^ and CB_2_^−/−^ mice)	[177]

**Table 5:** Overview of the putative inflammation-relevant therapeutic potential of cutaneous cannabinoid signaling.

Disease	Intervention	Level of Evidence	References
Sunburn	CB_1_&CB_2_ antagonism (?) ^1^	Cell culture, as well as KO-validated animal data	[87]
	TRPV4 antagonism	Cell culture, as well as KO-validated animal data	[86]
Allergic inflammation, atopic dermatitis (AD)	CB_1_ and/or CB_2_ agonism; FAAH-inhibition	Cell culture, as well as KO-validated animal data	[143,144,186,187,191,192]
	Topical CBC, CBCV, CBD, CBDV, Δ^8^-THCV, Δ^8^-THC, Δ^9^-THC	In vivo mouse data	[189]
	TRPV3 blockade or desensitization	Cell culture data	[119,121]
	*Echinacea purpurea*-derived alkylamides	Cell culture data and clinical trials	[199]
	PEA	Cell culture data, animal data and human clinical trials	[200,201,202,205]
	CB_2_ blockade (?) ^1^	Animal data	[214]
Excessive MC activity	PEA	Cell culture data	[226]
		Ex vivo dog skin organ culture data	[227]
	PEA-OXA (NAAA-inhibititor)	Animal data	[229]
	Activation of CB_1_	Cell culture data	[232,236,237]
		Ex vivo human HF and nasal polyp organ culture data	[245,246]
	Activation of CB_2_	Cell culture data	[232,236,242,243]
	Activation of PPARγ	Cell culture data	[242,243]
	TRPV3 blockade or desensitization ^2^	**Hypothesis** predicted based on animal data	[244]
PSO	CB_1_ activators (e.g., ACEA) via suppressing hyper-proliferation and K6 & K16 expression	Cell culture as well as ex vivo hSOC data	[174,175,182]
	NRIP1↓	Cell culture as well as NRIP1^−/−^ mice data	[277]
	TRIB3↓	Cell culture data	[279]
AD	TRPV1 antagonism	Ongoing phase II and III clinical trials	[293]
	TRPV3 antagonism or desensitization (candidate: CBGV) ^2^	Cell culture data	[119,121]
	FAAH-inhibition	Animal data	[144]
	CB_1_ activators	Animal data	[178]
	CB_2_ activators	Clinical study	[199]
		Animal data	[294]
	CB_2_ antagonists (?) ^1^	Animal data	[295]
	NAAA-inhibitors or PPARα agonists	Animal data	[297,298,299]
	PEA	Human clinical studies	[300,301]
	EMT-inhibition ^2^	**Hypothesis** based on cell culture data	[114]
	CBG, CBGV ^2^	**Hypothesis** based on cell culture data	[126]
SSc	TRPV4 blockade	Animal data	[324]
	CB_1_ antagonism	Animal data	[325]
		Cell culture data	[75]
	A_2A_ antagonism	Cell culture data	[75]
	CB_2_ activators	Cell culture data	[75]
		Animal data	[328]
		KO-validated animal data	[329,330]
	AJA (CB_2_ and PPARγ activator)	Cell culture data	[332]
		Animal data	[333]
		Completed phase II clinical trial, ongoing phase III trial	NCT02465437 NCT03398837 [319]
	VCE-004.3 (CB_2_ and PPARγ activator; CB_1_ antagonist)	Cell culture and animal data	[242]
	VCE-004.8/EHP-101 (CB_2_ and PPARγ activator)	Cell culture and animal data	[243,334]
	TRIB3↓	Animal data	[336]
	Pepcan-12 or THCV ^2^	**Hypothesis** based on the available data	[57,338]

^1^ Question marks indicate controversial data, which appear to contradict the majority of findings. ^2^ Note that well-controlled studies proving the efficiency of the indicated interventions are missing; however, in light of the available scarce data, they deserve systematic experimental exploration.

**Table 6:** Overview of the putative wound healing-relevant therapeutic potential of cutaneous cannabinoid signaling.

Condition	Intervention	Level of Evidence	References
Excisional wound	FAAH-inhibition and the subsequent elevation of N-acyl taurines	Animal data	[348]
Full-thickness wound	TRPV3 activation	Animal data	[354]
In vitro wound models	TRPV2 antagonism	Cell culture data	[355,356]
EB	Topical CBD	Case report of 3 patients	[183]

**Table 7:** Overview of the putative pruritus-relevant therapeutic potential of cutaneous cannabinoid signaling.

Condition	Intervention	Level of Evidence	References
Intractable cholestasis related itch	Dronabinol (5 mg at bedtime)	Pilot clinical data of 3 patients	[365]
Various itch models	CB_1_ activation	Animal data	[366,368]
		Human study	[370]
	CB_2_ activation	Animal data	[371]
	CB_2_ blockade (?) ^1^	Animal data	[295]
	FAAH- and/or MAGL-inhibition	Animal data	[372,375]
	NAAA-inhibition	Animal data	[297]
	PEA	Animal data	[202,299]
		Human clinical data	[300,377]

^1^ Question mark indicates controversial data, which appear to contradict the majority of findings.

**Table 8:** Overview of the putative anti-tumor potential of the cutaneous cannabinoid signaling.

Disease	Intervention	Level of Evidence	References
Melanoma	CB_1_ activation	Cell culture data	[390]
		Cell culture data	[392]
		Cell culture data	[394]
		Animal data	[394]
	CB_1_ antagonism (?) ^1^	Cell culture data	[382]
	CB_2_ activation	Cell culture data	[392]
		Animal data	[392]
		Cell culture data	[398]
	GPR18 blockade	Cell culture data	[397]
	GPR55 activation	Cell culture data	[390]
	PEA	Cell culture data	[391]
		Animal data	[391]
	β-caryophyllene	Cell culture data	[406]
		Animal data	[407]
	THC	Cell culture data	[392]
		Cell culture data and animal data	[409]
		Cell culture data	[410]
		Animal data	[410]
	THC+CBD	Cell culture data	[410]
		Animal data	[410]
Non-melanoma tumors	CB_1_ activation	Cell culture data	[133]
		Animal data	[133]
	CB_2_ activation	Cell culture data	[133]
		Animal data	[133]
	GPR55 blockade	Animal data	[415]
	CB_1_/CB_2_ blockade (?) ^1^	Animal data	[87]
	AEA administration	Cell culture data	[416,417,418]

^1^ Question marks indicate controversial data, which appear to contradict the majority of the findings.
